# Inferring putative ancient whole-genome duplications in the 1000 Plants (1KP) initiative: access to gene family phylogenies and age distributions

**DOI:** 10.1093/gigascience/giaa004

**Published:** 2020-02-11

**Authors:** Zheng Li, Michael S Barker

**Affiliations:** Department of Ecology and Evolutionary Biology, University of Arizona, 1041 E. Lowell St., Tucson, AZ 85721, USA

**Keywords:** whole-genome duplications, ancient whole-genome duplications, paleopolyploidy, transcriptomes, phylogenomics

## Abstract

**Background:**

Polyploidy, or whole-genome duplications (WGDs), repeatedly occurred during green plant evolution. To examine the evolutionary history of green plants in a phylogenomic framework, the 1KP project sequenced >1,000 transcriptomes across the Viridiplantae. The 1KP project provided a unique opportunity to study the distribution and occurrence of WGDs across the green plants. As an accompaniment to the capstone publication, this article provides expanded methodological details, results validation, and descriptions of newly released datasets that will aid researchers who wish to use the extended data generated by the 1KP project.

**Results:**

In the 1KP capstone analyses, we used a total evidence approach that combined inferences of WGDs from *Ks* and phylogenomic methods to infer and place 244 putative ancient WGDs across the Viridiplantae. Here, we provide an expanded explanation of our approach by describing our methodology and walk-through examples. We also evaluated the consistency of our WGD inferences by comparing them to evidence from published syntenic analyses of plant genome assemblies. We find that our inferences are consistent with whole-genome synteny analyses and our total evidence approach may minimize the false-positive rate throughout the dataset.

**Conclusions:**

We release 383,679 nuclear gene family phylogenies and 2,306 gene age distributions with *Ks* plots from the 1KP capstone paper. These resources will be useful for many future analyses on gene and genome evolution in green plants.

## Context

Ancient whole-genome duplications (WGDs), or paleopolyploidy, are found in the evolutionary history of many eukaryotes, especially in plants [1–6]. One of the major discoveries of the early era of plant genome sequencing was the observation of ancient WGDs in most sequenced plant genomes [2, 7]. Despite progress on understanding the distribution of WGDs across the phylogeny of green plants, many lineages have remained unstudied for lack of data. The 1000 plants project (1KP) [8] sequenced the transcriptomes of 1,173 plant species from across the green plant phylogeny. These newly sequenced data provided crucial new genomic data for previously under- or unsampled lineages of green plants. The 1KP capstone analyses inferred putative WGDs and assessed their frequency and distribution across the green plant tree of life. As an accompaniment to the 1KP capstone paper [8], here we provide detailed methodology of the total evidence approach used in the 1KP ancient WGD analyses. To better demonstrate our approach, we present analyses of 2 different sets of WGDs as walk-through examples. We also compared the consistency of our WGD inferences with whole-genome synteny analyses. By providing further methodological insight, results validation, and descriptions of data released from the 1KP ancient WGD analyses, this companion to the 1KP capstone paper should aid other researchers who are interested in reusing these data from the 1KP project.

## Methods

The expansive phylogenetic sampling of the 1KP provided an opportunity to infer putative WGDs and assess their frequency and distribution across the green plant tree of life. To survey potential WGDs, we used a total evidence approach to infer and place putative ancient WGDs in the 1KP capstone phylogeny. WGDs were inferred from age distributions of gene duplications by analyzing transcriptomes of single species with the DupPipe pipeline [9]. To place inferred WGDs from *Ks* plots onto the species phylogeny, we compared the median paralog divergence (*Ks*) of putative WGD peaks to the divergence of orthologs among species across the phylogeny [9]. We also used phylogenomic analyses and simulations of WGDs using MultiAxon Paleopolyploidy Search (MAPS) [3, 10] to corroborate the inferences and phylogenetic placements of the putative ancient WGDs. Here we provide details of our analyses as well as *Ks* plots that represent each major lineage and 2 walk-through examples from our 1KP capstone analyses to demonstrate our total evidence approach. Finally, we evaluate our inferences of WGDs by comparing them with evidence from published syntenic analyses of plant genome assemblies.

### Data release for DupPipe analyses of ancient WGDs

**Figure 1:**
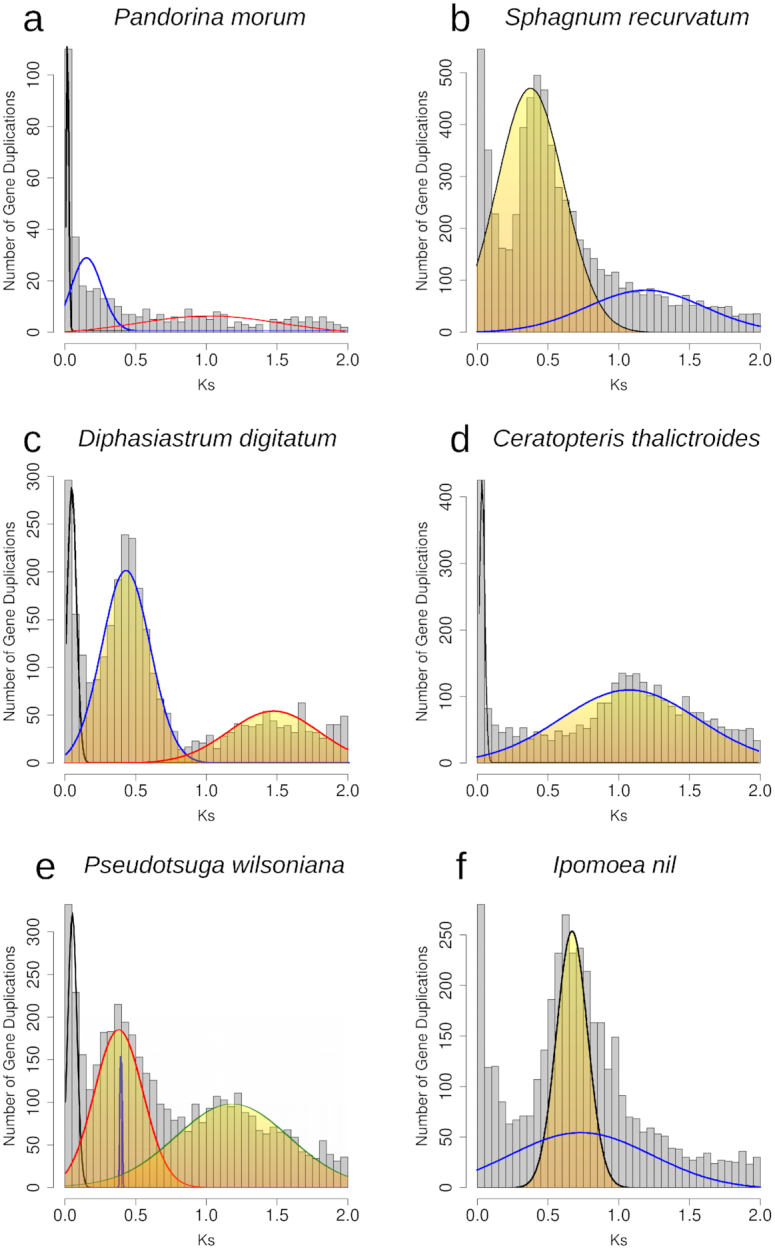
Histograms of the age distribution of gene duplications (*Ks* plots) with mixture models of inferred WGDs for **(a)***Pandorina morum* (green algae), no inferred WGD peak. **(b)***Sphagnum recurvatum* (moss), inferred WGD peak median *Ks* = 0.38. **(c)***Diphasiastrum digitatum* (lycophyte), inferred WGD peak median *Ks* = 0.42, 1.62. **(d)***Ceratopteris thalictroides* (fern), inferred WGD peak median *Ks* = 1.08. **(e)***Pseudotsuga wilsoniana* (gymnosperm), inferred WGD peak median *Ks* = 0.38, 1.18. **(f)***Ipomoea nil* (angiosperm) inferred WGD peak median *Ks* = 0.66. Histogram x-axis scale is *Ks* 0–2. The mixture model distributions consistent with inferred ancient WGDs are highlighted in yellow.

**Figure 2:**
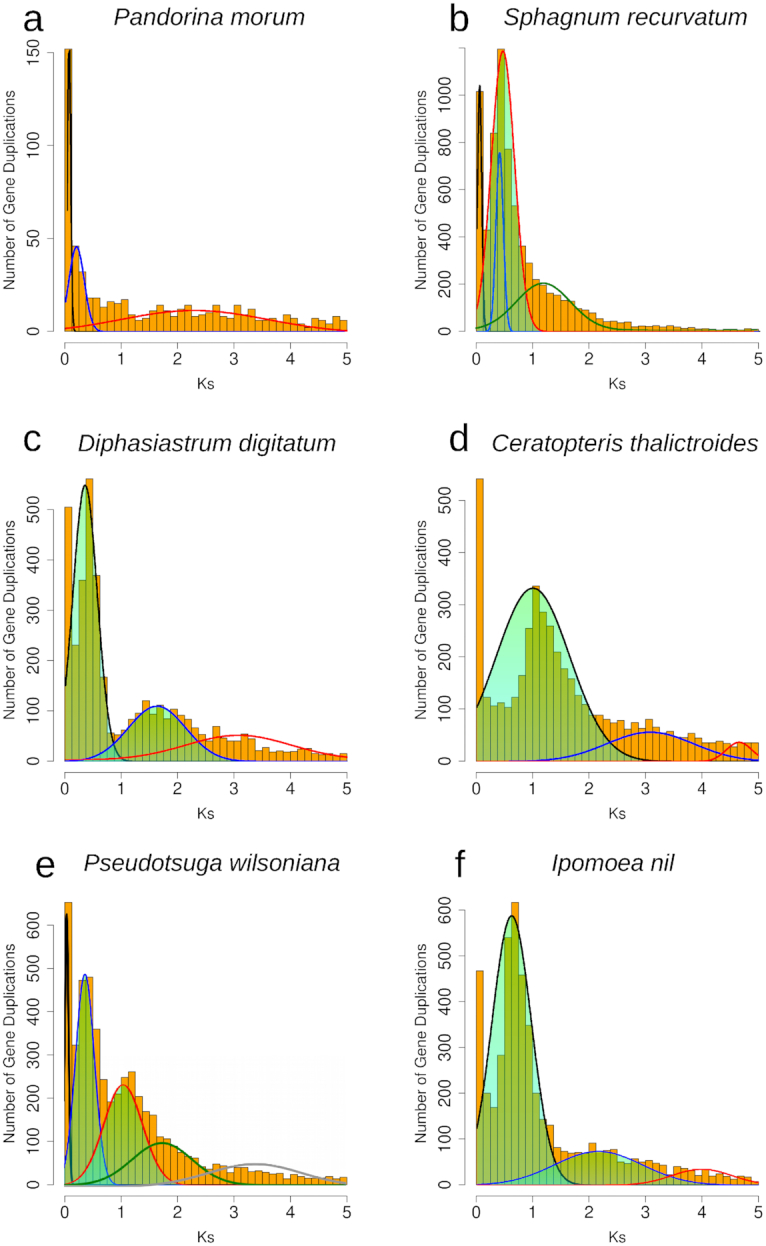
Histograms of the age distribution of gene duplications (*Ks* plots) with mixture models of inferred WGDs for **(a)***Pandorina morum* (green algae), no inferred WGD peak. **(b)***Sphagnum recurvatum* (moss), inferred WGD peak median *Ks* = 0.38. **(c)***Diphasiastrum digitatum* (lycophyte), inferred WGD peak median *Ks* = 0.42, 1.62. **(d)***Ceratopteris thalictroides* (fern), inferred WGD peak median *Ks* = 1.08, 3.07. **(e)***Pseudotsuga wilsoniana* (gymnosperm), inferred WGD peak median *Ks* = 0.38, 1.18. **(f)***Ipomoea nil* (angiosperm) inferred WGD peak median *Ks* = 0.66, 2.15. Histogram x-axis scale is *Ks* 0–5. The mixture model distributions consistent with inferred ancient WGDs are highlighted in green.

For each transcriptome of the 1KP [11], we used the DupPipe pipeline to construct gene families and estimate the age of gene duplications [9]. We identified duplicate pairs as sequences that demonstrate 40% sequence similarity over ≥300 base pairs from a discontinguous MegaBLAST [12, 13]. We translated DNA sequences and identified reading frames by comparing the Genewise (Genewise, RRID:SCR_015054) [14] alignment to the best-hit protein from a collection of proteins from 25 plant genomes from Phytozome (Phytozome, RRID:SCR_006507) [15]. For each analysis, we used protein-guided DNA alignments to align our nucleic acid sequences while maintaining reading frame. Best-hit proteins are paired with each gene at a minimum cut-off of 30% sequence similarity over ≥150 sites. Gene families are then constructed by single-linkage clustering. We then estimated synonymous divergence (*Ks*) using PAML (PAML, RRID:SCR_014932) [16] with the F3 × 4 model for each node in the gene family phylogenies. A recent study has shown that estimating the node *Ks* values for duplicates from gene family trees rather than pairwise comparisons of paralogs can reduce error in estimating *Ks* values of duplication events and has a significant effect on the resolution of WGD peaks [17]. In this project, we used the approach described in Tiley et al. 2018 [17]. Previous analyses also indicate that there is reasonable power to infer WGDs in *Ks* plots when paralog divergences are *Ks* < 2. Saturation and other errors accumulate at paralog divergences of *Ks* > 2 and can create false signals of WGDs and make distinguishing true WGDs from the background a fraught task [17, 18]. We followed the recommendations of these studies in all of our 1KP *Ks* plot inferences. Although we plotted and presented 2 sets of histograms with x-axis scales of *Ks* = 2 and *Ks* = 5 to assess WGDs at different resolutions (Figs 1, 2), we did not identify peaks with *Ks* > 2 as potential WGDs without other data available (e.g., synteny or phylogenomic evidence). Note that this means the rate of substitution in a lineage limits the depth of time at which we can reliably infer the presence or absence of putative WGDs. Here, we provided the 1,153 raw output files from the DupPipe pipeline and the 2,306 *Ks* plots generated in these analyses. Each raw output file is a tab-delimited text file containing the node *Ks* value for each duplication. Gene annotation from the *Arabidopsis thaliana* gene ontology is provided. All files are available in bitbucket and GigaDB [19].

To identify significant features in the gene age distributions that may correspond to WGDs, we used 2 statistical tests: Kolmogorov-Smirnov (K-S) goodness-of-fit tests and mixture models. We first identified taxa with potential WGDs by comparing their paralog ages to a simulated null distribution without ancient WGDs using a K-S goodness-of-fit test [20]. For taxa with evidence for a significant peak relative to the null, we then used a mixture model implemented in the mixtools R package [21] to identify significant peaks of gene duplication consistent with WGDs and estimate their median *Ks* values (Figs 1 and 2). These approaches have been used to infer WGDs in *Ks* plots in many species that were subsequently corroborated by syntenic analyses of whole-genome sequences [20, 22–24]. There is a recent trend in the community of authors simply surveying the *Ks* plots of single species without a model or statistical inference to infer a WGD (e.g., [25–28]). By using these 2 statistical tests, our results have been more rigorously evaluated than many recent studies of WGDs.

To visually demonstrate our gene age distribution approach, we provide example *Ks* plots for 4 major lineages across the green plant phylogeny. In the green alga *Pandorina morum*, the K-S test indicated that the paralog age distribution was significantly different than a simulated null. However, we do not observe any peaks of duplication consistent with the expected signature of an ancient WGD from the 2 sets of histograms (Figs 1a, 2a). In other land plant examples, the K-S test also found that paralog age distributions were significantly different than null simulations (*P* < 0.001). In the bryophyte example, we observed single peaks of duplication consistent with an ancient WGD in the *Ks* plots of each species (*Sphagnum recurvatum*, median *Ks* = 0.38, Figs 1b, 2b).In the lycophyte, fern, gymnosperm, and angiosperm examples, we observed 2 peaks of duplication consistent with 2 rounds of putative ancient WGD in each species. The mixtools mixture models estimated that these putative WGD peaks have median *Ks* of 0.42 and 1.62 in *Diphasiastrum digitatum* (Figs 1c, 2c), median Ks of 1.08 and 3.07 in *Ceratopteris thalictroides*(Figs 1d, 2d), median *Ks* values of 0.38 and 1.18 in *Pseudotsuga wilsoniana* (Figs 1e, 2e), and median *Ks* values of 0.66 and 2.15 in *Ipomoea nil* (Figs 1f, 2f).

### Estimating orthologous divergence

To place putative WGDs in the context of lineage divergence, we estimated the synonymous divergence of orthologs among pairs of species that may bracket the phylogenetic position of a WGD in our sampled taxa. Orthologs were identified as reciprocal best blast hits in pairs of transcriptomes using the RBH Ortholog pipeline [9]. This pipeline uses protein-guided DNA alignments to align our nucleic acid sequences while maintaining reading frame. The pairwise synonymous (*Ks*) divergence for each pair of orthologs is then estimated using PAML with the F3 × 4 model [16]. The mean and median ortholog synonymous divergences were recorded and compared to the synonymous divergence of inferred paleopolyploid peaks estimated by the mixture model. If the median synonymous divergence of WGD paralogs was younger than the median synonymous divergence of orthologs, WGDs were interpreted to have occurred after lineage divergence. Similarly, if the synonymous divergence of WGD paralogs was older than the ortholog synonymous divergence, then we interpreted those WGDs as shared by those taxa. By comparing paralog and ortholog synonymous divergences, we placed inferred ancient WGDs in a phylogenetic context. To better demonstrate this ortholog divergence analysis, we provide an example using a putative WGD inferred in the ancestry of the Pinaceae in section for walk-through examples.

### Data release for MAPS analyses of ancient WGDs

We used MAPS, a gene tree topology sorting algorithm [3, 10], to confirm the placement of ancient WGDs that may be shared by ≥3 species. MAPS uses a given species tree to filter collections of nuclear gene trees for subtrees consistent with relationships at each node in the species tree. For each MAPS analysis, gene families were clustered using OrthoFinder (OrthoFinder, RRID:SCR_017118) [29] with reciprocal protein BLAST (blastp) searches using an E-value of 10e−5 as a cut-off. Gene families were clustered using the default parameters of OrthoFinder. We filtered the gene family clusters to include only gene families that contained ≥1 gene copy from each taxon. We constructed alignments and phylogenies for each gene family using PASTA [30]. For each gene family phylogeny, we ran PASTA (PASTA, RRID:SCR_008770) until we reached 3 iterations without an improvement in likelihood score using a centroid breaking strategy. Within each iteration of PASTA, we constructed subset alignments using MAFFT (MAFFT, RRID:SCR_011811) [31] and used Muscle (Muscle, RRID:SCR_011812) [32] for merging these subset alignments and RAxML (RAxML, RRID:SCR_006086) [33] for tree estimation. The parameters for each software package were the default options for PASTA. We used the best-scoring PASTA tree for each multi-species nuclear gene family to collectively estimate the numbers of shared gene duplications on each branch of the given species. To maintain sufficient gene tree numbers to infer ancient WGDs, we used collections of gene trees for 6–8 taxa for each MAPS analysis. The entire collection of 383,679 nuclear gene family phylogenies and alignments generated for all MAPS analyses are provided. The compressed files are named by the corresponding MAPS analysis in the 1KP capstone manuscript. The -aln folder contains the alignment files for each gene tree analyzed by MAPS, whereas the -tre folder contains the gene tree files. The readme in each compressed file contains the taxon identifiers and species names used in each MASP analysis. All files are available in bitbucket and GigaDB [19].

**Figure 3:**
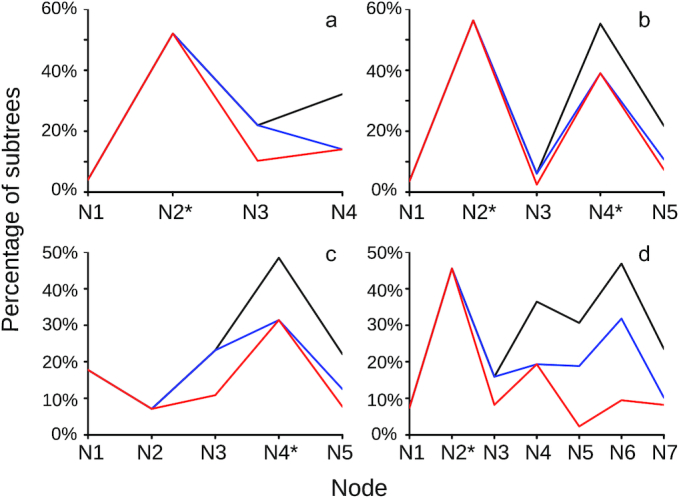
Increasing taxon occupancy decreases the inflation of mapped duplications towards the root of the species tree in MAPS. The black line represents the MAPS result without the minimum taxon requirement. The blue line represents the MAPS results with a 35% minimum taxa requirement. The red line represents the MAPS results with a 45% minimum taxa requirement. N1 corresponds to the tip node, and the last node (e.g., N4 in **(a)**) corresponds to the root node. Asterisk represents nodes associated with inferred WGDs. **(a)** 1KP MAPS result of eudicot ancient hexaploidy event; N2 represents the node associated with this paleohexaploidy event. See MAPS E21 in the One Thousand Plant Transcriptomes Initiative (2019) [8] for details. **(b)** N2 represents the node associated with an inferred Pinaceae WGD; N4 represents the node associated with the inferred seed plant WGD. See Fig. 4c, d for the phylogeny. **(c)** N4 represents node associated with the paleohexaploidy event shared by most Compositae; see Fig. 5a for the phylogeny. **(d)** N4 represents node associated with the Heliantheae ancient WGD; see Fig. 5b for the phylogeny.

We selected taxa for our MAPS analyses to minimize potential mapping errors at the tips and roots of species trees. Gene tree error may create a bias that causes more gene losses to map at the tips and more gene duplications to map to roots in gene tree reconciliation analyses [34]. Although there is not a general solution to this problem, we used 2 different approaches in our MAPS analyses to minimize the impact of this known issue. First, we expect the tips and roots of our MAPS analyses to have much higher duplication mapping error. Given that the numbers of subtrees at the tips and roots may be skewed, we have lower confidence in estimates at the tip and root nodes compared to the number of mapped duplications in the center of our MAPS phylogenies. For this reason, we aimed to place the focal WGD test node in the middle of the phylogeny being examined. Second, we implemented an option in MAPS to increase taxon occupancy in the gene trees by requiring a minimum number of ingroup taxa be present in each subtree [3]. Based on previous work [35] and balancing the number of trees retained in our analyses, we used a minimum 45% ingroup taxa requirement in our MAPS analyses. If this minimum ingroup taxa number requirement is not met for a gene tree, it will be filtered out and excluded from our analysis. As we discussed in Li et al. 2018 [3], requiring higher taxon occupancy greatly reduced the bias of mapping duplications to older nodes of the phylogeny as observed by Hahn (2007) [34] and led to less inflated estimates of duplications on deeper nodes (Fig. 3).

As genomic data have expanded, methods for inferring WGDs from phylogenetic analyses have matured over time to include more formal approaches for assessing WGDs. The increased taxon sampling present in larger datasets has allowed the field to begin analyzing genomic data from multiple related species that may have a shared WGD in their ancestry. Some early phylogenomic approaches simply used a hard cut-off based on numbers or percentages of gene trees to label an episode of gene duplication a putative WGD [26]. Although many WGDs may be inferred because of large changes in duplication numbers across a phylogeny, gene duplications vary across the phylogeny because of changes in branch length and variation in gene birth and death rates. We introduced simulations and statistical analyses in MAPS to address some of the issues associated with the phylogenomic inference of ancient WGDs [3]. Ancient WGDs are inferred in 2 steps in the MAPS framework. We first develop a null simulation of the number of expected duplications on each branch of our species tree based on a range of estimated background gene birth and death rates. The null simulation used gene birth and death rates estimated from each tree using WGDgc as described in Rabier et al. [36], and used the GuestTreeGen program from GenPhyloData [37] to generate simulated gene trees as described in Li et al. (2018) [3]. This null simulation accounts for variation in the number and percent of gene duplications associated with branch length and background birth/death rates among the sampled taxa. Significant bursts above this null indicate a deviation from the background birth and death rate as expected for episodic events like WGDs. We used the Fisher exact test to compare our observed MAPS results to the null simulations and identify significant episodes of duplication. All nodes are compared against the null model to identify significant episodes of gene duplication across a species tree. Once these significant episodes of gene duplication are identified, we used a second set of gene tree simulations to assess whether they were consistent with a WGD. Again, we used the Fisher exact test to compare our observed numbers of duplications to the number of shared duplications expected with a WGD at a particular location in the phylogeny. If these increases in gene duplications were caused by a WGD, then we expect the numbers of shared gene duplications among extant taxa to be consistent with these positive simulations. By using these simulations and statistical methods, MAPS explicitly accounts for the number of duplications expected on branches of different lengths within species trees and provides a statistical test to assess whether an episode of duplication is consistent with a potential ancient WGD.

It should be emphasized that we used a total evidence approach to infer WGDs in the 1KP capstone project. We combined evidence from single-species *Ks* plots, pairwise ortholog divergence analyses, and multispecies MAPS analyses to identify ancient episodes of gene duplication consistent with WGDs and place them on our species tree. For example, we did not call a WGD based only on evidence from a MAPS analysis. In the few cases where the results of our different inference approaches conflicted, we relied on the weight of evidence from a majority of analyses and, if available, other analyses from the literature to infer a putative WGD. These were mostly cases where inferences from *Ks* plots, ortholog comparisons, and the previous literature agreed but MAPS did not. In these cases, we recognized the event as a significant burst of gene duplication and indicated this in Supplemental text and tables, and labeled as blue squares on the ED WGD Phylogeny Figure [8]. These events may be WGDs that should be analyzed in subsequent analyses with new data or methods.

## Walk-through examples

**Figure 4:**
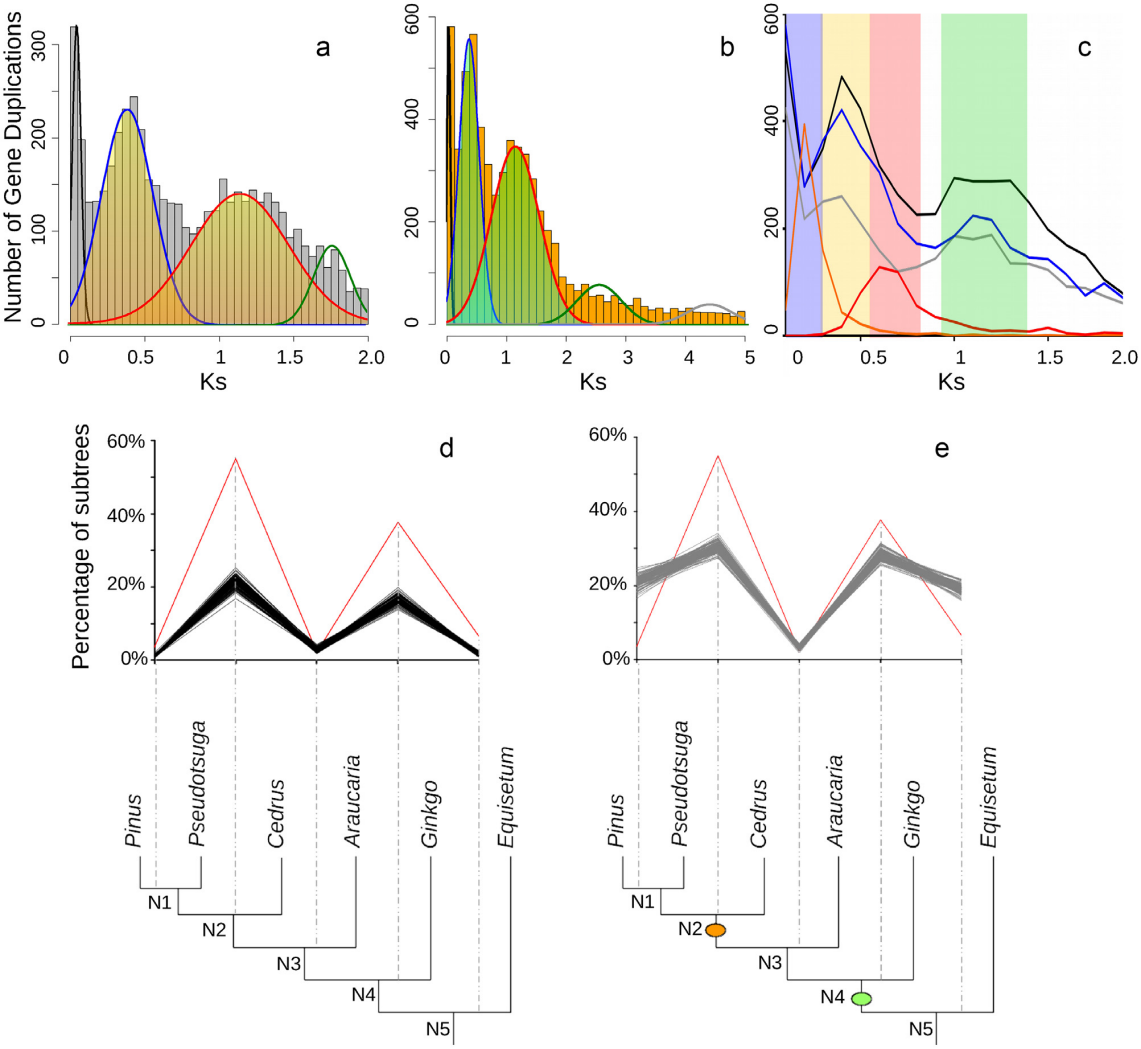
Histograms of the age distribution of gene duplications (*Ks* plots), ortholog divergences, and MAPS results for the Pinaceae ancient WGD. **(a)** and **(b)** Histograms of the age distribution of gene duplications (*Ks* plots) with mixture models of inferred WGDs for *Pseudotsuga wilsoniana* (gymnosperm), inferred WGD peak median *Ks* = 0.38 (95% CI, 0.371–0.386) and 1.18 (95% CI, 1.163–1.195). **(a)** Histogram x-axis scale is *Ks* 0–2. The mixture model distributions consistent with inferred ancient WGDs are highlighted in yellow. **(b)** Histogram x-axis scale is *Ks* 0–5. The mixture model distributions consistent with inferred ancient WGDs are highlighted in green. **(c)** Combined *Ks* plot of the gene age distributions of *P. wilsoniana* (blue), *Pinus radiata* (black), *Cedrus libani* (gray), and ortholog divergences of *Pinus* vs *Cedrus* (orange) and *Cedrus* (Pinaceae) vs *Cephalotaxus* (Cephalotaxaceae) (red). The median peaks for these plots are highlighted. *Pinus radiata* (black), inferred WGD peak medians at *Ks* = 0.37 (95% CI, 0.365–0.380) and 1.16 (95% CI, 1.142–1.172). *C. libani* (gray), inferred WGD peak medians at *Ks* = 0.33 (95% CI, 0.316–0.336) and 1.08 (95% CI, 1.061–1.099). **(d)** and **(e)** MAPS results from observed data, null, and positive simulations on the associated phylogeny. **(d)** Percentage of subtrees that contain a gene duplication shared by descendant species at each node, results from observed data (red line), 100 resampled sets of null simulations (black lines). **(e)** Percentage of subtrees that contain a gene duplication shared by descendant species at each node, results from observed data (red line), and positive simulations (gray lines). The orange oval corresponds to the location of an inferred WGD in Pinaceae. The green oval corresponds to the location of an inferred WGD in seed plants.

**Figure 5:**
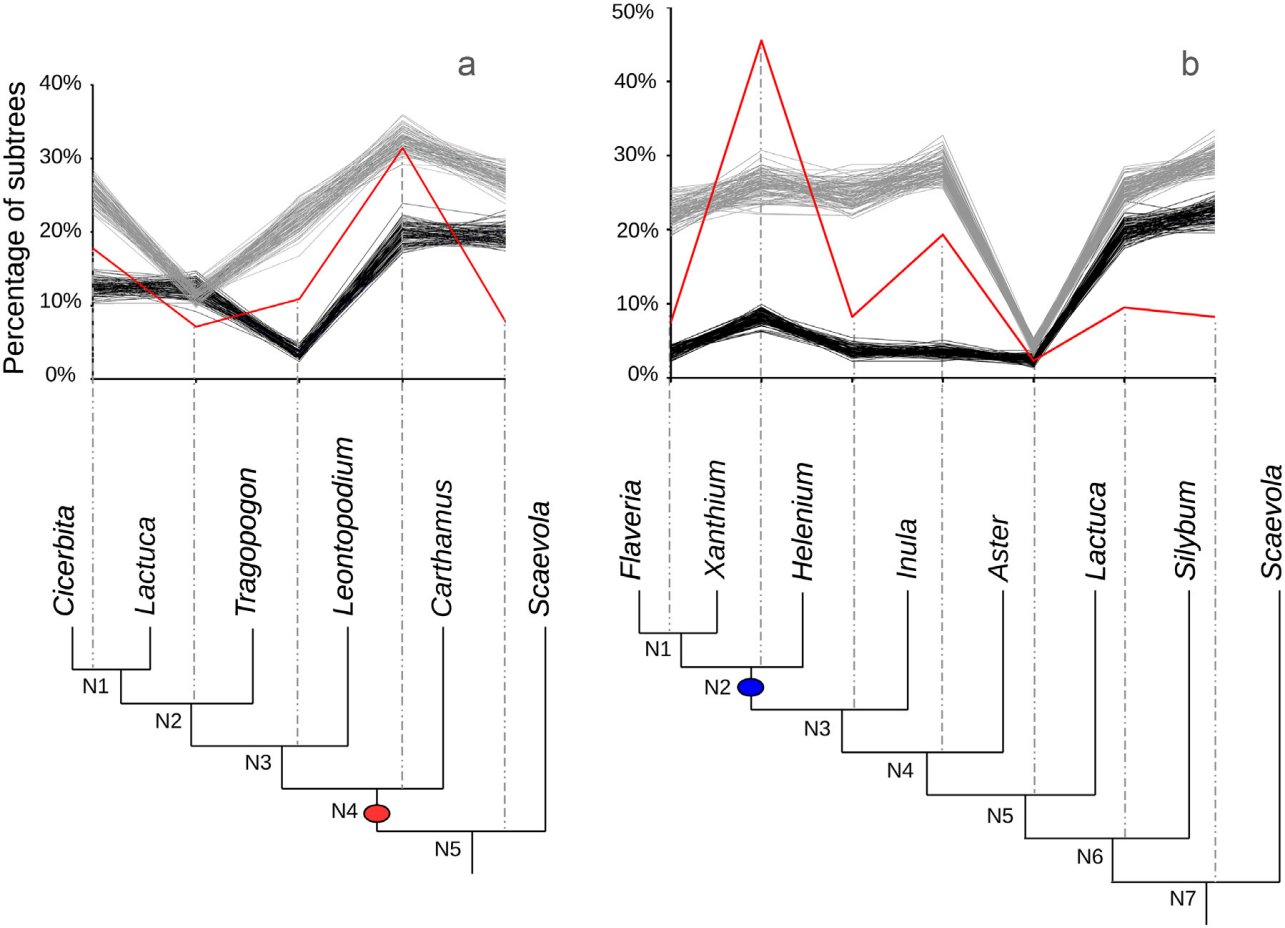
Asteraceae MAPS results from observed data, null, and positive simulations on the associated phylogeny. **(a)** Percentage of subtrees that contain a gene duplication shared by descendant species at each node, results from observed data (red line), 100 resampled sets of null simulations (black lines), and positive simulations (gray lines). The red oval corresponds to the paleohexaploidy event in the Compositae. **(b)** Percentage of subtrees that contain a gene duplication shared by descendant species at each node, results from observed data (red line), 100 resampled sets of null simulations (black lines), and positive simulations (gray lines). The blue oval corresponds to the Heliantheae ancient WGD.

To better demonstrate our approach for inferring ancient WGDs, we selected 2 examples from the 1KP analyses as walk-throughs. We chose the Pinaceae and Compositae ancient WGD analyses as examples (Figs 4 and 5) because these analyses represent different scales and complexities of duplication events. Previous analyses have found evidence for 2 rounds of WGD in the history of the Pinaceae [10], including a potential WGD in the ancestry of all seed plants [10, 38]. However, other analyses have questioned the placement and/or existence of significant bursts of gene duplication in these lineages [39, 40]. In contrast, the Compositae walk-through example has no conflict among studies but is a complex nested paleohexaploidy in the ancestry of one of the largest families of flowering plants [41, 42]. Inferring the location of the nested WGDs that comprise the paleohexaploidy, while also distinguishing other WGDs in these data, is a potentially challenging task for transcriptome-based phylogenomic analyses. Below, we walk through our results for these examples and explain how we arrived at our inferences of a WGD (or not). It should be noted that we conducted a similar level of analysis and decision-making process in the inference of all 244 putative WGDs in the 1KP capstone analysis.

Consistent with previous research [10, 38], we observed evidence for ≥2 rounds of duplication in the ancestry of the Pinaceae. We observed 2 peaks of duplication consistent with 2 rounds of ancient WGDs in the history of 3 Pinaceae genera (*Pinus, Pseudotsuga*, and *Cedrus*; Fig. 4). Recent peaks of duplication in species of *Pinus, Pseudotsuga*, and *Cedrus* have a median *Ks* ∼ 0.3 (Fig. 4a–c), older than their ortholog divergences (*Ks* ∼ 0.18; Fig. 4c). These ortholog divergence analyses suggest that the younger putative WGD in the 3 species is most likely shared by all Pinaceae. However, this putative WGD is not likely shared by other conifers because the ortholog divergence of the Pinaceae to other conifers is nearly twice the paralog divergence of the putative WGD. For example, the ortholog divergence of members of the Pinaceae relative to members of the Cephalotaxaceae is *Ks* ∼ 0.6 (Fig. 4c), consistent with this duplication event occurring after the divergence of these conifer families. The older peaks observed in *Pinus, Pseudotsuga*, and *Cedrus* have a median *Ks* ∼ 1 (Fig. 4c), most likely shared by all seed plants but more recent than the divergence of seed plants and ferns (*Ks* ∼ 3, estimated in 1KP capstone project).

As described above, we further assessed the nature of phylogenetic position of these putative WGDs using MAPS. We selected species of *Pinus, Pseudotsuga*, and *Cedrus* to represent Pinaceae in this MAPS analyses. We also selected species of *Araucaria* and *Ginkgo* to represent other gymnosperms, and species of *Equisetum* and *Selaginella* were used as outgroups. For the null simulations, we first simulated 3,000 gene trees using the mean background gene duplication rate (λ) and gene loss rate (μ). We then randomly resampled 1,000 trees without replacement from the total pool of gene trees 100 times to provide a measure of uncertainty of the percentage of subtrees at each node (Fig. 4d). At nodes corresponding to N1, N2, N4, and N5, we observed significantly more shared duplications than expected compared to the null simulations (*P* < 0.01) (Fig. 4d). For positive simulations, we incorporated a WGD at nodes N1, N2, N4, and N5 and simulated gene trees using the same methods described above. At the node representing the most recent common ancestor of Pinaceae (N2) and the node representing the most recent common ancestor of gymnosperms (N4), we identified an episodic burst of shared gene duplication that is statistically consistent with our positive simulations of WGDs (Fig. 4e). The results from our comparison to the null and positive simulations are consistent with those from *Ks* plots and ortholog divergence analyses described above, as well as those of our previous study in gymnosperms [10]. These results and another MAPS analysis in the 1KP capstone project (MAPS D1) show evidence consistent with a putative ancient WGD shared among all Pinaceae and another putative WGD that likely occurred in the ancestry of seed plants [8].

In addition to our analyses with the 1KP capstone dataset, other analyses have also inferred a putative WGD in the ancestry of all seed plants [10, 38, 39] and in the ancestry of different conifer families [10]. Consistent with our previous analyses [10], the relatively dense phylogenetic sampling of the 1KP allowed us to confirm that the putative seed plant WGD is not shared with monilophytes. A recent study proposed that cycads and *Ginkgo* might have shared another round of ancient WGD(s) [27]. However, other analyses in the 1KP capstone (MAPS D1 and related ortholog divergence analyses) using 3 species of cycads, *Ginkgo, Amborella*, and outgroups reject this hypothesis. Instead, we find evidence that the signature detected by Roodt et al. (2017) [27] in cycads and *Ginkgo* is most likely the putative seed plant WGD (One Thousand Plant Transcriptomes Initiative 2019 [8]). In the 1KP and previous research [10], we also found evidence for other putative ancient WGDs in the ancestry of some families of conifers, including the Pinaceae as described above. Using whole-genome data from *Ginkgo biloba, Picea abies*, and *Pinus taeda*, a recent study does not find evidence in both *Ks* plots and phylogenomic analyses for the Pinaceae WGD [39]. The absence of a putative Pinaceae WGD peak in their *Ks* plot is possibly due to the quality of the genome assembly and annotation, or the scaling of their *Ks* plot, which may obscure the peaks we observed in all Pinaceae taxa. In the 1KP capstone project, we consistently observed 2 peaks of gene duplication consistent with putative WGDs in all *Ks* plots from the 14 species of Pinaceae analyzed. Only 1 conifer species, *Picea abies*, was included in the analysis by Zwaenepoel and Van de Peer [39]. It is possible that the lack of support for the Pinaceae WGD is due to the limited sampling of conifers because they [39] demonstrated that taxon sampling can have a significant impact on WGD inference with taxon-dependent support for the well-established eudicot hexaploidy [24, 43–46]. Given the aforementioned evidence from *Ks* plots, ortholog divergence, and MAPS analyses, our inference and placement of the putative Pinaceae and seed plants WGDs is currently the best explanation for these large-scale gene duplication events. Future studies with new data, especially with higher-quality gymnosperm genome assemblies, are needed to test these hypothesized WGDs.

To further demonstrate our total evidence approach to resolve complex ancient WGDs, we provide a walk-through of our analyses of ancient WGDs in the Asteraceae. We previously inferred 2 rounds of ancient WGD consistent with a paleohexaploidy in the ancestry of the Compositae [22, 41, 42]. The paleohexaploid nature of this WGD was later supported by synteny analyses of the sunflower and other Compositae genomes [47–49]. Given the great phylogenetic depth of sampling in the 1KP project and our introduction of a new statistical test for inferring WGD in MAPS [3] since our previous analysis, we re-evaluated the ancient WGDs with 2 new MAPS analyses and new data in the 1KP capstone (One Thousand Plant Transcriptomes Initiative 2019 [8]). In 1 of the MAPS analyses (Fig. 5a), we selected species of *Cicerbita, Lactuca, Tragopogon, Leontopodium*, and *Carthamus* to represent the Compositae. Data from *Scaevola* and *Menyanthes* were used as outgroups. Our new analyses with the 1KP data confirmed the phylogenetic position of the paleohexaploidy in the ancestry of the Compositae (Fig. 5a). In the second analysis (Fig. 5b), we used the expanded phylogenetic sampling of the 1KP to more precisely locate an additional WGD in the ancestry of the Heliantheae previously inferred by *Ks* plots and ortholog divergence analyses [22] and synteny [48]. We selected species of *Flaveria, Xanthium*, and *Helenium* to represent the tribe Heliantheae, and species of *Inula* and 4 other genera as outgroups. Our analysis of new 1KP data confirmed the location of the Heliantheae WGD with a significant peak of gene duplication consistent with a simulated WGD in the history of all Heliantheae sampled (Fig. 5). Our Compositae analyses in the 1KP allowed us to re-evaluate established WGDs using data from newly sampled taxa and more precisely locate these in the phylogeny. More than 100 of the 1KP WGDs were previously inferred, and the expanded sampling of the 1KP dataset allowed us to more precisely place them as we did here in the Compositae.

## Evaluation of WGD inferences

**Figure 6:**
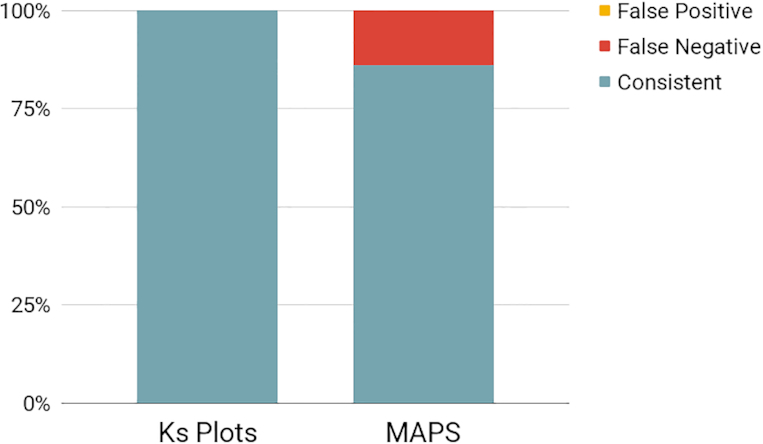
Consistency of the 1KP *Ks* and MAPS inferences of WGD with results from published synteny analyses of plant genomes. Consistent results are represented by blue, and false-negative results, by red. There were no false-positive results in our inferences of WGDs compared to those from published synteny analyses.

To evaluate our WGD inferences from the 1KP capstone project [8], we compared the consistency of our inferences with whole-genome synteny analyses. Although limited in placing WGDs on a phylogeny because of the relatively low phylogenetic sampling of assembled genomes, synteny analysis using high-quality genomes is generally considered the best approach for confirming an ancient WGD [45, 50]. We compared the results of our *Ks* and MAPS analyses with analyses of WGDs from published synteny analyses of plant genomes (Fig. 6, Supplementary Table 1). Overall, we were able to make 65 comparisons of our *Ks* plot inferences and 43 comparisons of our MAPS phylogenomic analyses to syntenic analyses. Our inferences of WGDs with *Ks* plots and ortholog divergences were 100% consistent with syntenic analyses from either the same species or a close relative (Fig. 6, Supplementary Table 1). Despite a perception that *Ks* plots are difficult to interpret or unreliable, a recent study found that *Ks* plot analyses using best practices, as we did here, are highly robust [17]. Thus, the high consistency of our *Ks* plot inferences of WGDs with published genome analyses is not unexpected. We observed slightly lower consistency of our MAPS phylogenomic inferences of WGDs. Across the 43 synteny comparisons, we observed no false-positive results but did observe 6 false-negative results (Fig. 6, Supplementary Table 1). This tendency of our phylogenomic method towards false-negative results and “missing” established WGDs is a known issue. There are cases of well-established WGDs going undetected with different phylogenomic analyses, including *At-α* [51] and the eudicot gamma hexaploidy [39]. Although MAPS and other phylogenomic approaches are often viewed as more rigorous than single-species approaches like *Ks* plots and synteny, these approaches are sensitive to a variety of parameters including gene tree sample size, taxon composition, gene tree occupancy, variation in branch lengths, variation in gene birth/death rates, and variation in gene retention and loss patterns across the phylogeny, to name a few. Notably, we did not observe false-positive inferences of WGDs with MAPS, and there do not appear to be reports of false-positive inferences in the literature from other phylogenomic methods. However, false signals of large bursts of gene duplication, potentially on the scale consistent with a WGD, could be created by incomplete lineage sorting and quirks of gene tree reconciliation [34]. To minimize the potential biases of these types of phylogenomic methods in the 1KP capstone project, we aimed to use a total evidence approach that combined inferences across *Ks* plots, ortholog divergence analyses, and MAPS phylogenomic analyses to infer WGDs. Considering that we observed no false-positive results and high consistency of our *Ks* and MAPS analyses with syntenic results, we think that our survey of WGDs across the phylogeny of green plants is reasonably robust and the combined approach minimized false-positive results. We expect that some of the 244 WGDs we inferred may move location or be merged as more data become available, and emphasize that the 138 newly inferred WGDs should be treated as hypotheses until confirmed with further data to corroborate the nature and precise timing of these large-scale gene duplication events.

## Availability of Supporting Data and Materials

Supporting data are available in the GigaScience GigaDB repository [19]. Source code and sequencing data of the 1KP project are described in more detail in another accompanying Data Note [11].

All supporting data are also available in bitbucket at https://bitbucket.org/barkerlab/1kp/src/master/.

## Additional Files

**Supplementary Table 1:** Survey of consistency of the 1KP *Ks* and MAPS inferences of WGD with results from published synteny analyses of plant genomes. This table contains data for 65 comparisons of 1KP *Ks* plot inferences and 43 comparisons of MAPS phylogenomic analyses to plant genome synteny analyses.

giaa004_GIGA-D-19-00294_Original_Submission

giaa004_GIGA-D-19-00294_Revision_1

giaa004_GIGA-D-19-00294_Revision_2

giaa004_Response_to_Reviewer_Comments_Original_Submission

giaa004_Response_to_Reviewer_Comments_Revision_1

giaa004_Reviewer_1_Report_Original_Submission Yafei Mao -- 8/24/2019 Reviewed

giaa004_Reviewer_1_Report_Revision_1 Yafei Mao -- 12/15/2019 Reviewed

giaa004_Reviewer_2_Report_Original_Submission Tao Ma -- 9/4/2019 Reviewed

giaa004_Supplemental_Table

## Abbreviations

1KP: 1,000 Plants project; BLAST: Basic Local Alignment Search Tool; K-S: Kolmogorov-Smirnov; MAPS: MultitAxon Paleopolyploidy Search; NSF: National Science Foundation; PAML: Phylogenetic Analysis by Maximum Likelihood; RAxML: Randomized Axelerated Maximum Likelihood; RBH: Reciprocal Best Hit; WGD: whole-genome duplication.

## Competing Interests

The authors declare that they have no competing interests.

## Funding

The 1KP initiative was funded by the Alberta Ministry of Advanced Education and Alberta Innovates AITF/iCORE Strategic Chair (RES0010334) to Gane Ka-Shu Wong, Musea Ventures, The National Key Research and Development Program of China (2016YFE0122000), The Ministry of Science and Technology of the People’s Republic of China (2015BAD04B01/2015BAD04B03), the State Key Laboratory of Agricultural Genomics (2011DQ782025) and the Guangdong Provincial Key Laboratory of core collection of crop genetic resources research and application (2011A091000047). We thank Gane Ka-Shu Wong for providing the 1KP funding. Genome duplication analyses were supported by US National Science Foundation (NSF) grants IOS-1339156 and EF-1550838 to M.S.B..

## Authors' Contributions

Z.L. and M.S.B. performed data analyses; Z.L. and M.S.B. wrote the manuscript.
