# Supplementary material for: Inferring putative ancient whole-genome duplications in the 1000 Plants (1KP) initiative: access to gene family phylogenies and age distributions
Source: Gigascience. 2020 Feb 11;9(2):giaa004. doi: 10.1093/gigascience/giaa004 (PMC7011446; doi:10.1093/gigascience/giaa004)
Supplement: giaa004_GIGA-D-19-00294_Revision_1 [file giaa004_giga-d-19-00294_revision_1.pdf]

GigaScience

Inferring putative ancient whole genome duplications in the 1000 Plants (1KP) initiative: access to gene family phylogenies and age distributions

--Manuscript Draft--

**Manuscript Number:**	GIGA-D-19-00294R1
**Full Title:**	Inferring putative ancient whole genome duplications in the 1000 Plants (1KP) initiative: access to gene family phylogenies and age distributions
**Article Type:**	Data Note
**Funding Information:**	Division of Integrative Organismal Systems (IOS-1339156) — Dr. Michael S Barker<br>Division of Emerging Frontiers (EF-1550838) — Dr. Michael S Barker
**Abstract:**	Polyploidy or whole genome duplications (WGDs) repeatedly occurred during green plant evolution. To examine the evolutionary history of green plants in a phylogenomic framework, the 1KP project sequenced over 1000 transcriptomes across the Viridiplantae. The 1KP project provided a unique opportunity to study the distribution and occurrence of WGDs across the green plants. As an accompaniment to the capstone publication, this paper provides expanded methodological details, results validation, and descriptions of newly released data sets that will aid researchers that wish to use the extended data generated by the 1KP project. In the 1KP capstone analyses, we used a total evidence approach that combined inferences of WGDs from Ks and phylogenomic methods to infer and place 244 putative ancient WGDs across the Viridiplantae. Here, we provide an expanded explanation of our approach by describing our methodology and walkthrough examples. We also evaluated the consistency of our WGD inferences by comparing them to evidence from published syntenic analyses of plant genome assemblies. We find that our inferences are consistent with whole genome synteny analyses and our total evidence approach may minimize the false positive rate throughout the data set. Given these resources will be useful for many future analyses on gene and genome evolution in green plants, we release 383,679 nuclear gene family phylogenies and 2,306 gene age distributions with Ks plots from the 1KP capstone paper.
**Corresponding Author:**	Zheng Li<br>University of Arizona<br>Tucson, AZ UNITED STATES
**Corresponding Author Secondary Information:**	
**Corresponding Author's Institution:**	University of Arizona
**Corresponding Author's Secondary Institution:**	
**First Author:**	Zheng Li
**First Author Secondary Information:**	
**Order of Authors:**	Zheng Li<br>Michael S Barker
**Order of Authors Secondary Information:**	
**Response to Reviewers:**	Dear Scott,<br><br>We thank you and both reviewers for the thorough reviews and suggestions to improve our manuscript. We have made a number of revisions to the manuscript to address the specific concerns of reviewers to better demonstrate the goal of this accompaniment paper. These included revising the abstract and adding a new Context section. We also revised and included detailed methodology for the DupPipe pipeline. We feel the suggested revisions have improved the quality of the manuscript and we thank the

Powered by Editorial Manager® and ProduXion Manager® from Aries Systems Corporation

reviewer #1 for bring up many valid suggestions in our analyses. Overall, our revised manuscript is clearer on demonstrate the value of this accompaniment. Below, we respond to each reviewer comment and describe in detail how we have addressed the issues identified and the corresponding revisions to our manuscript. Our responses are typed here and revisions to the manuscript itself are highlighted in yellow.

Zheng Li

Reviewer reports:

Reviewer #1: Whole-genome duplications (WGDs) play an important role in the evolution of plants and animals. Investigating the WGDs in plants with 1KP genomic data provides us a big picture of genome evolution in green plants. Although lots of publications have addressed this question, Li's manuscript provides a systematical analysis for the 1KP dataset and then it opens a road for further WGDs' research in the future. Therefore, I recommend this manuscript to publish in the journal.
We greatly appreciated reviewer #1’s suggestions and comments. Please see our detailed response below.

However, I have a few comments on the analysis and the manuscript's structure.

Major comments:

1. What is the main point in this manuscript? "Building a total evidence approach for WGDs' research" or "Investigating WGDs in green plants with 1KP dataset using a total evidence approach".
This manuscript is one of the accompaniment papers to the 1KP Nature capstone paper. All these accompaniment papers aim to provide detailed methodology, validation, and provide a guide to describe the complex data of the accompanying Nature article. In this accompaniment manuscript, we aim to provide provide expanded methodology of the WGD analyses by using walkthrough examples, provide validation to our ancient WGD inferences, and release relevant data and other materials from the 1KP ancient WGD analyses.

The abstract makes me feel that this manuscript's main point on "Investigating WGDs in green plants with 1KP dataset using a total evidence approach", yet the main text makes me feel that the manuscript describes "a total evidence approach" for WGDs' research and using 10KP as a system.
We appreciate the reviewer’s comment on the abstract. This is a very good point. We have revised our abstract and added a new Context section. We hope this makes more clear for the goal of this accompaniment paper.

For my understanding, "a total evidence approach" has been applied and described in Jiao's paper and Mao's previous paper (Jiao, Yuannian, et al; Nature; 2011; Mao, Yafei, et al; iScience; 2019). I recommend authors to re-organize the main text to show the point that "Investigating WGDs in green plants with 1KP dataset using a total evidence approach" with INTRODUCTION, METHODS, RESULTS, AND DISCUSSION. This would make this manuscript clear and have much impact on plant WGDs' research in the future.
As we described the purpose of this manuscript above, this is accompaniment papers to the 1KP Nature capstone paper. The format suggested by the reviewer is for a Research Article. In this manuscript, we follow the standard and format for a Data Note in GigaScience.

Plus, a personal taste, I would like to use another word to replace "a total evidence approach", what is the "total evidence"? We might have other methods to infer WGDs and do better in the future. (if authors would like to use "a total evidence approach" in the revised manuscript, I have no problem with it).
We appreciate the reviewer’s comment on this part. As we discussed in this manuscript, we agree with the reviewer that future improvement in methods and data will better infer ancient WGDs across the tree of life. For the wording of ‘a total evidence approach’, this term is commonly used in the field of Phylogenetics and Systematics (For example: Ronquist et al. 2012 Syst. Bio.; Arrigo et al. 2013 AJB;

Powered by Editorial Manager® and ProduXion Manager® from Aries Systems Corporation

Gomes-Silva et al. 2017 Cladistics). It describes the philosophical principle that we will evaluate use multiple lines of evidence to test a hypothesis. In Systematics for example, a total evidence approach requires one to bring in molecular, morphological, and as many data as possible to understand the evolutionary history of organisms. In the 1KP Nature capstone analysis, we use the total evidence approach to infer ancient WGDs by using gene age distribution analysis from transcriptome of single species, orthologous divergence estimation between species to place WGDs, MAPS phylogenomic analyses from transcriptomes of multiple species to infer and place WGDs, and syntenic evidence from published plant genomes to compare with our inferences. We believe it is appropriate to call combining these different lines of evidence as 'a total evidence approach' for the 1KP Nature capstone analysis.

2. In abstract, "we release 383,679 nuclear gene family phylogenies and 2,306 gene age distribution (Ks) plots from the 1KP capstone paper." Could you release original data rather plots?
Yes, we added the original output files into the bitbucket repository.

In the method 1.1 "DupPipe analyses of WGDs from transcriptomes of single species", could you release the all orthogroups data which were generated by DupPipe pipeline? As well, in the method 1.3 "MAPS analyses of WGDs from transcriptomes of multiple species", could you release the orthogroups data which were generated by OrthoFinder?
The orthogroups data are available in the 1KP bitbucket repository.
https://bitbucket.org/barkerlab/1kp/src/master/
For the DupPipe pipeline, all gene family output can now be found in the original output files. For MAPS, all the orthogroups data that are used in MAPS which were generated by OrthoFinder can be found in the tar files in the 1KP bitbucket repository. These tar files are organized by each MAPS analysis. For each gene family, an alignment file and a gene family phylogeny in newick format can be downloaded from the bitbucket repository.

Curious, why do authors use two different methods (DupPipe and OrthoFinder) to generate orthogroups for Ks and MAPS analysis, respectively? (I do not think the methods will have a big impact on the final results, but I'd like to know more about the authors' experimental design.)
The DupPipe pipeline uses reciprocal best hit followed by single linkage clustering to generate gene families and then uses PAML to calculate the node Ks. Using the reciprocal best hit approach to construct gene families from a single transcriptome or genome is commonly used and highly efficient (Tatusov et al. 1997 Science, Bork et al. 1998 J. Mol. Biol.). As we demonstrated recently (Tiley et al. 2018 GBE) and discussed previously (Barker et al. 2008 MBE), using the node Ks values from gene families rather than the pairwise Ks value reduces error in estimating the Ks values of duplication events and improves the resolution of Ks peaks (Tiley et al. 2018 GBE). The DupPipe analyses are focussed on single species. In MAPS, we search for ancient WGDs across genomic data from multiple species. These species might be highly diverged, for example, mosses and seed plants diverged 600 Mya. Given that orthogroups need to be clustered in a much larger dataset (multiple transcriptomes or genomes from many species) and sequences that may be highly divergent, we use a more sophisticated gene family clustering algorithm such as OrthoMCL (Li et al, 2003) or OrthoFinder (Emms et al. 2015) to address these challenges.

3. I am not an expert for statistical tests, I have no more comments on statistical test in this manuscript. All look good for me by far.
We appreciate the reviewer's comment on our statistical tests.

4. In method 1.3, authors used RAxML to reconstruct gene trees, and then I am wondering whether authors used Partitionfinder to select best-fit partitioning schemes as input for RAxML to avoid gene tree bias. If so, authors should mention this in the method section. If not, I recommend the authors to perform this analysis. Alternatively, authors could use IQ-TREE to perform best-fit partitioning schemes finding and tree reconstruction together automatically. My program TREEasy (https://github.com/MaoYafei/TREEasy) might be helpful for a large amount of tree reconstruction with IQ-TREE. However, authors should choose the easier way for themselves to solve this problem, no need to use TREEasy as a must.

Powered by Editorial Manager® and ProduXion Manager® from Aries Systems Corporation

We used RAxML for constructing gene trees because it is implemented in PASTA, and used 'PROTWAGCAT' for AA model which is the default setting in PASTA. Partitionfinder is written before the data explosion in phylogenomics (Lanfear et al, 2012 MBE). It is considered to be too slow for large genomic and transcriptomic datasets (Lanfear et al. 2017 MBE). We will consider using Partitionfinder 2 (Lanfear et al. 2017 MBE) or TREEasy in the future.

5. About Figures, I recommend authors to consider "integrate" Figure 1 and Figure 2 together. If so, it would be more concise for readers. Moreover, If I were authors, I would like to use a table to present Figure 6 (you already have it in your supplementary file).
We thank the reviewer's comment on the Figures. For Figure 1 and Figure 2, we believe it is more appropriate to have two different figure panels. As we stated in text "we plotted and presented two sets of histograms with x-axis scales of Ks = 2 and Ks = 5 to assess WGDs at different resolutions (Fig. 1, Fig. 2)". We also posted these two different sets of histograms on the bitbucket repository. It is clearer to show these are two different sets of histograms by having two separate figures. It also makes each plot bigger. This helps visualizing the details of the Ks plots. For Figure 6, the current figure provides a simple and clear visualization of the consistent, false positive, and false negative rates. Changing Figure 6 into a table will likely lose the simple visualization and make it harder to process the same information. For readers that are interested in any details of this validation, one can look up or search related information in the SI Table 1.

6. About re-organization of the manuscript as I suggested above, if authors would like to do it, I also suggest that authors could mention WGDs in other clades rather than plants. This might attract more readers who are not studying WGDs in plants.
We have added this at the beginning of the context section and cited relevant papers.

Minor comments:

1. 1KP_WGD_phylogeny.pdf in (https://bitbucket.org/barkerlab/1kp/src/master/1KP_WGD_phylogeny.pdf) might be truncated. See the top of PDF, some lineages did not show in the pdf file.
We checked the pdf file. This file is not truncated for us on at least two different computers.

2. In "Walk-through examples", like "a median Ks ~0.3". Could you also put the 95% CI there? Also, could you put the line number in the revised manuscript? Sometimes, it is really hard to point out sentences.
We thank the reviewer's comment. For "a median Ks ~0.3", we want to state that both Pinus, Pseudotsuga, and Cedrus has a WGD peak with a median Ks around 0.3. This pattern can be easily observed in Fig. 4c. We also highlighted it in yellow in Fig. 4c. We added the median Ks value estimated from the mixture model and 95% CI for both Pinus, Pseudotsuga, and Cedrus as requested in the Fig. 4c legend. All median Ks values for WGD peaks inferred by mixture models are also provided in the 1KP capstone paper as a searchable spreadsheet.

We add the version of the revised manuscript with line numbers.

3. In "This tendency of our phylogenomic method towards false negatives and "missing" established WGDs is a known issue. There are cases of well-established WGDs going undetected with different phylogenomic analyses, including At-[43]". "AT-[]" might have some formatting error here.
This is a format converting error. We corrected it to 'At-α'.

4. In "In this project, we used the approach described in Tiley et al. 2018. Previous analyses". Is "in this study" better than "In this project"?
We change 'in this project' to 'in the 1KP capstone project' to avoid any confusion.

5. Tiley et al. 2018 paper also mentioned that inferred WGDs are more robust when Ks<1 rather than Ks<2. But, I think Ks<2 is acceptable. Maybe it is worth to mention this in the discussion.

Powered by Editorial Manager® and ProduXion Manager® from Aries Systems Corporation

	We agree with the reviewer. We stated in the manuscript that there is reasonable power to infer ancient WGDs when Ks < 2 in this manuscript, please see line 87-98. We also cited Vanneste et al. 2013 and Tiley et al. 2018 which discussed this issue in depth.<br><br>6. Using transcriptomes to infer WGD with age distribution sometime is dangerous because transcriptomes assembly mistakes or incomplete transcriptomes assembly would lead to loss of WGD signals. Authors should mention this in the discussion.<br>We mentioned this in the Evaluation of WGD inferences section. As we stated in line 377-379 ‘Despite a perception that Ks plots are difficult to interpret or unreliable, a recent study found that Ks plots analyses using best practices, as we did here, are highly robust’. Here, our evaluation of Ks plot WGD inferences were 100% consistent with published genome analyses. Stating transcriptomes assembly mistakes or incomplete transcriptomes assembly would lead to loss of WGD signals is also misleading. Potential issues or mistakes in assembly can also apply to whole genome sequencing.<br><br>Reviewer #2: In this manuscript, the authors showed the detailed information about the 1KP data and the pipline they used in the WGD inference. As it is a companion paper, I think it is very necessary and useful to have such a description for the data anslysis.<br><br>However, I found that the explanation of the pipline is not very clear for the DupPipe analyses. The authors should have a more detailed description, especially for readers who are not familar with the DupPipe pipeline.<br>We greatly appreciate reviewer #2 comments and suggestions. We revised the section for the DupPipe analyses by adding methodological details. Please also be aware that DupPipe is a published pipeline and we cited the original papers that describe the pipeline in more detail (Barker et al. 2008 MBE and Barker et al. 2010 Evo. Bioinfo.)<br><br>such as:<br><br>How they construct gene families using the transcriptomes from only one species?<br>The DupPipe pipeline uses a reciprocal best blast hit and single linkage clustering to construct gene families from a single genome or transcriptome. Using the reciprocal best hit approach to construct gene families from a single transcriptome or genome is commonly used and highly efficient (Tatusov et al. 1997 Science, Bork et al. 1998 J. Mol. Biol.). We analyzed these gene family clusters with PAML to calculate the node Ks rather than the pairwise Ks value (Barker et al. 2010 Evo. Bioinfo.). This approach can reduce error in estimating Ks values of duplication events and improve the resolution of Ks peaks (Tiley et al. 2018 GBE). We have added the details for gene family clustering in the DupPipe section. Please see line 74-82. Please notice that DupPipe is a published pipeline, and we cited the original papers that describe the pipeline in more detail (Barker et al. 2008 MBE and Barker et al. 2010 Evo. Bioinfo.)<br><br>and How they identify peaks in the distribution of KS values?<br>The second paragraph of the section ‘2.1 DupPipe analyses of WGDs from transcriptomes of single species’ describes how we identify significant features in the gene age distributions that may correspond to WGDs. We used two statistical tests: Kolmogorov–Smirnov goodness of fit tests and mixture models. 1) By using the using a K–S goodness of fit test, we first identified taxa with potential WGDs by comparing their paralog ages to a simulated null distribution without ancient WGDs. 2) For taxa with evidence for a significant peak relative to the null, we used a mixture model implemented in the mixtools R package to identify significant peaks of gene duplication consistent with WGDs. Please see line 104-116.
**Additional Information:**	
**Question**	**Response**
Are you submitting this manuscript to a special series or article collection?	No
**Experimental design and statistics**	Yes

Powered by Editorial Manager® and ProduXion Manager® from Aries Systems Corporation

Full details of the experimental design and statistical methods used should be given in the Methods section, as detailed in our Minimum Standards Reporting Checklist. Information essential to interpreting the data presented should be made available in the figure legends.<br><br>Have you included all the information requested in your manuscript?	
**Resources**<br><br>A description of all resources used, including antibodies, cell lines, animals and software tools, with enough information to allow them to be uniquely identified, should be included in the Methods section. Authors are strongly encouraged to cite Research Resource Identifiers (RRIDs) for antibodies, model organisms and tools, where possible.<br><br>Have you included the information requested as detailed in our Minimum Standards Reporting Checklist?	Yes
**Availability of data and materials**<br><br>All datasets and code on which the conclusions of the paper rely must be either included in your submission or deposited in publicly available repositories (where available and ethically appropriate), referencing such data using a unique identifier in the references and in the "Availability of Data and Materials" section of your manuscript.<br><br>Have you have met the above requirement as detailed in our Minimum Standards Reporting Checklist?	Yes

Powered by Editorial Manager® and ProduXion Manager® from Aries Systems Corporation

Manuscript
Click here to view linked References
Click here to access/download;Manuscript;Li&Barker_1KP_gigascience_revisi

Inferring putative ancient whole genome duplications in the 1000 Plants (1KP) initiative: access to gene family phylogenies and age distributions

Zheng Li (liz7@email.arizona.edu)
Michael S Barker (msbarker@email.arizona.edu)

Department of Ecology and Evolutionary Biology, University of Arizona, Tucson, AZ 85721

Abstract

Polyploidy or whole genome duplications (WGDs) repeatedly occurred during green plant evolution. To examine the evolutionary history of green plants in a phylogenomic framework, the 1KP project sequenced over 1000 transcriptomes across the Viridiplantae. The 1KP project provided a unique opportunity to study the distribution and occurrence of WGDs across the green plants. As an accompaniment to the capstone publication, this paper provides expanded methodological details, results validation, and descriptions of newly released data sets that will aid researchers that wish to use the extended data generated by the 1KP project. In the 1KP capstone analyses, we used a total evidence approach that combined inferences of WGDs from Ks and phylogenomic methods to infer and place 244 putative ancient WGDs across the Viridiplantae. Here, we provide an expanded explanation of our approach by describing our methodology and walkthrough examples. We also evaluated the consistency of our WGD inferences by comparing them to evidence from published syntenic analyses of plant genome assemblies. We find that our inferences are consistent with whole genome synteny analyses and our total evidence approach may minimize the false positive rate throughout the data set. Given these resources will be useful for many future analyses on gene and genome evolution in green plants, we release 383,679 nuclear gene family phylogenies and 2,306 gene age distributions with Ks plots from the 1KP capstone paper.

Keywords

Whole genome duplications (WGDs), ancient WGDs, paleopolyploidy, transcriptomes, phylogenomics

1. Context

Ancient whole genome duplications (WGDs) or paleopolyploidy is found in

the evolutionary history of many eukaryotes, especially in plants [1–6]. One of the major discoveries of the early era of plant genome sequence was the observation of ancient WGDs in most sequenced plant genomes [2,7]. Despite progress on understanding the distribution of WGDs across the phylogeny of green plants, many lineages have remained unstudied for lack of data. The 1000 plants (1KP) project [8] sequenced the transcriptomes of 1,173 plant species from across the green plant phylogeny. These newly sequenced data provided crucial new genomic data for previously under- or unsampled lineages of green plants. The 1KP capstone analyses inferred putative WGDs and assessed their frequency and distribution across the green plant tree of life. As an accompaniment to the 1KP capstone paper [8], here we provide detailed methodology of the total evidence approach used in the 1KP ancient WGD analyses. To better demonstrate our approach, we present analyses of two different sets of WGDs as walkthrough examples. We also compared the consistency of our WGD inferences with whole genome synteny analyses. By providing further methodological insight, results validation, and descriptions of data released from the 1KP ancient WGD analyses, this companion to the 1KP capstone paper should aid other researchers that are interested in reusing these data from the 1KP project.

2. Methods

The expansive phylogenetic sampling of the 1KP provided an opportunity to infer putative WGDs and assess their frequency and distribution across the green plant tree of life. To survey potential WGDs, we used a total evidence approach to infer and place putative ancient WGDs in the 1KP capstone phylogeny. WGDs were inferred from age distributions of gene duplications by analyzing transcriptomes of single species with the DupPipe pipeline [9]. To place inferred WGDs from Ks plots onto the species phylogeny, we compared the median paralog divergence (*Ks*) of putative WGD peaks to the divergence of orthologs among species across the phylogeny [9]. We also employed phylogenomic analyses and simulations of WGDs using MultitAxon Paleopolyploidy Search(MAPS) [3,10] to corroborate the inferences and phylogenetic placements of the putative ancient WGDs. Here we provide details of our analyses as well as Ks plots that represent each major lineages and two walkthrough examples from our 1KP capstone analyses to demonstrate our total evidence approach. Finally, we evaluate our inferences of WGDs by comparing them with evidence from published syntenic analyses of plant genome assemblies.

2.1 Data release for DupPipe analyses of ancient WGDs

For each transcriptome, we used the DupPipe pipeline to construct gene families and estimate the age of gene duplications [9]. We identified duplicate pairs are as sequences that demonstrate 40% sequence similarity over at least 300 base pairs from a discontinguous MegaBLAST[11,12]. We translated DNA sequences and identified reading frames by comparing the Genewise [13] alignment to the best-hit protein from a collection of proteins from 25 plant genomes from Phytozome [14]. For each analysis, we used protein-guided DNA alignments to align our nucleic acid sequences while maintaining reading frame. Best hit proteins are paired with each gene at a minimum cutoff of 30% sequence similarity over at least 150 sites. Gene families are then constructed by single-linkage clustering. We then estimated synonymous divergence (*Ks*) using PAML [15] with the F3X4 model for each node in the gene family phylogenies. A recent study has shown that estimating the node Ks values for duplicates from gene family trees rather than pairwise comparisons of paralogs can reduce error in estimating Ks values of duplication events and has a significant impact on the resolution of WGD peaks [16]. In this project, we used the approach described in Tiley et al. 2018. Previous analyses also indicate that there is reasonable power to infer WGDs in Ks plots when paralog divergences are Ks < 2. Saturation and other errors accumulate at paralog divergences of Ks > 2 and can create false signals of WGDs and make distinguishing true WGDs from the background a fraught task [16,17]. We followed the recommendations of these studies in all of our 1KP Ks plot inferences. Although we plotted and presented two sets of histograms with x-axis scales of Ks = 2 and Ks = 5 to assess WGDs at different resolutions (Fig. 1, Fig. 2), we did not identify peaks with Ks > 2 as potential WGDs without other data available (e.g., synteny or phylogenomic evidence). Note that this means the rate of substitution in a lineage limits the depth of time at which we can reliably infer the presence or absence of putative WGDs. Here, we provided the 1153 raw output files from the DupPipe pipeline and the 2,306 Ks plots generated in these analyses. Each raw output file is a tab de-limited text file containing the node Ks value for each duplication. Gene annotation from the *Arabidopsis thaliana* gene ontology is provided. All files are available here: https://bitbucket.org/barkerlab/1kp/src/master/.

To identify significant features in the gene age distributions that may correspond to WGDs, we used two statistical tests: Kolmogorov–Smirnov goodness of fit tests and mixture models. We first identified taxa with potential WGDs by comparing their paralog ages to a simulated null

distribution without ancient WGDs using a K–S goodness of fit test [18]. For taxa with evidence for a significant peak relative to the null, we then used a mixture model implemented in the mixtools R package [19] to identify significant peaks of gene duplication consistent with WGDs and estimate their median Ks values (Fig. 1, Fig. 2). These approaches have been used to infer WGDs in Ks plots in many species that were subsequently corroborated by syntenic analyses of whole genome sequences [18,20–22]. There is a recent trend in the community of authors simply surveying the Ks plots of single species without a model or statistical inference to infer a WGD (e.g., [23–26]). By using these two statistical tests, our results have been more rigorously evaluated than many recent studies of WGDs.

To visually demonstrate our gene age distribution approach, we provide example Ks plots for four major lineages across the green plant phylogeny. In the green alga *Pandorina morum*, the K-S test indicated that the paralog age distribution was significantly different than a simulated null. However, we do not observe any peaks of duplication consistent with the expected signature of an ancient WGD from the two sets of histograms (Fig. 1a, Fig. 2a). In other land plant examples, the K-S test also found paralog age distributions were significantly different than null simulations (p = 0). In the bryophyte and fern examples, we observed single peaks of duplication consistent with an ancient WGD in the Ks plots of each species (*Sphagnum recurvatum,* median Ks = 0.3814, Fig. 1b & 2b; *Ceratopteris thalictroides,* median Ks = 1.0793, Fig. 1d & 2d). In the lycophyte, gymnosperm, and angiosperm examples, we observed two peaks of duplication consistent with two rounds of putative ancient WGD in each species. The mixtools mixture models estimated that these putative WGD peaks have median Ks of 0.4247 and 1.6229 in *Diphasiastrum digitatum* (Fig. 1c, Fig. 2c), median Ks values of 0.3724 and 1.1572 in *Pinus radiata* (Fig. 1d, Fig. 2d), and median Ks values of 0.6646 and 2.1532 in *Ipomoea nil* (Fig. 1e, Fig. 2e).

2.2 Estimating orthologous divergence

To place putative WGDs in the context of lineage divergence, we estimated the synonymous divergence of orthologs among pairs of species that may bracket the phylogenetic position of a WGD in our sampled taxa. Orthologs were identified as reciprocal best blast hits in pairs of transcriptomes using the RBH Ortholog pipeline [9]. This pipeline uses protein-guided DNA alignments to align our nucleic acid sequences while maintaining reading frame. The pairwise synonymous (*Ks*) divergence for each pair of orthologs is then estimated using PAML with the F3X4 model [15]. The mean and

median ortholog synonymous divergences were recorded and compared to the synonymous divergence of inferred paleopolyploid peaks estimated by the mixture model. If the median synonymous divergence of WGD paralogs was younger than the median synonymous divergence of orthologs, WGDs were interpreted to have occurred after lineage divergence. Similarly, if the synonymous divergence of WGD paralogs was older than the ortholog synonymous divergence, then we interpreted those WGDs as shared by those taxa. By comparing paralog and ortholog synonymous divergences, we placed inferred ancient WGDs in a phylogenetic context. To better demonstrate this ortholog divergence analysis, we provide a walk through example using a putative WGD inferred in the ancestry of the Pinaceae in section 2.

2.3 Data release for MAPS analyses of ancient WGDs

We used MAPS, a gene tree topology sorting algorithm [3,10], to confirm the placement of ancient WGDs that may be shared by at least three species. MAPS uses a given species tree to filter collections of nuclear gene trees for subtrees consistent with relationships at each node in the species tree. For each MAPS analysis, gene families were clustered using OrthoFinder [27] with reciprocal protein BLAST (blastp) searches using an E-value of 10e-5 as a cutoff. Gene families were clustered using the default parameters of OrthoFinder. We filtered the gene family clusters to include only gene families that contained at least one gene copy from each taxon. We constructed alignments and phylogenies for each gene family using PASTA [28]. For each gene family phylogeny, we ran PASTA until we reached three iterations without an improvement in likelihood score using a centroid breaking strategy. Within each iteration of PASTA, we constructed subset alignments using MAFFT [29], employed Muscle [30] for merging these subset alignments, and RAxML [31] for tree estimation. The parameters for each software package were the default options for PASTA. We used the best scoring PASTA tree for each multi-species nuclear gene family to collectively estimate the numbers of shared gene duplications on each branch of the given species. To maintain sufficient gene tree numbers to infer ancient WGDs, we used collections of gene trees for six to eight taxa for each MAPS analysis. The entire collection of 383,679 nuclear gene family phylogenies and alignments generated for all MAPS analyses are provided. The compressed files are named by the corresponding MAPS analysis in the 1KP capstone manuscript. The -aln folder contains the alignment files for each gene tree analyzed by MAPS, whereas the -tre folder contains the gene tree files. The readme in each compressed file contains the taxon identifiers

and species names used in each MASP analysis. All files are available here: https://bitbucket.org/barkerlab/1kp/src/master/.

We selected taxa for our MAPS analyses to minimize potential mapping errors at the tips and roots of species trees. Gene tree error may create a bias that causes more gene losses to map at the tips and more gene duplications to map to roots in gene tree reconciliation analyses [32]. Although there is not a general solution to this problem, we used two different approaches in our MAPS analyses to minimize the impact of this known issue. First, we expect the tips and roots of our MAPS analyses to have much higher duplication mapping error. Given that the numbers of subtrees at the tips and roots may be skewed, we have lower confidence in estimates at the tip and root nodes compared to the number of mapped duplications in the center of our MAPS phylogenies. For this reason, we aimed to place the focal WGD test node in the middle of the phylogeny being examined. Secondly, we implemented an option in MAPS to increase taxon occupancy in the gene trees by requiring a minimum number of ingroup taxa be present in each subtree [3]. Based on previous work [33] and balancing the number of trees retained in our analyses, we used a minimum 45% ingroup taxa requirement in our MAPS analyses. If this minimum ingroup taxa number requirement is not met for a gene tree, it will be filtered out and excluded from our analysis. As we discussed in Li et al. 2018, requiring higher taxon occupancy greatly reduced the bias of mapping duplications to older nodes of the phylogeny as observed by Hahn (2007) and led to less inflated estimates of duplications on deeper nodes (Fig. 3).

As genomic data has expanded, methods for inferring WGDs from phylogenetic analyses have matured over time to include more formal approaches for assessing WGDs. The increased taxon sampling present in larger datasets has allowed the field to begin analyzing genomic data from multiple related species that may have a shared WGD in their ancestry. Some early phylogenomic approaches simply used a hard cutoff based on numbers or percentages of gene trees to label an episode of gene duplication a putative WGD [24]. Although many WGDs may be inferred because of large changes in duplication numbers across a phylogeny, gene duplications vary across the phylogeny because of changes in branch length and variation in gene birth and death rates. We introduced simulations and statistical analyses in MAPS to address some of the issues associated with the phylogenomic inference of ancient WGDs [3]. Ancient WGDs are inferred

in two steps in the MAPS framework. We first develop a null simulation of the number of expected duplications on each branch of our species tree based on a range of estimated background gene birth and death rates. The null simulation used gene birth and death rates estimated from each tree using WGDgc as described in [34], and used the GuestTreeGen program from GenPhyloData [35] to generate simulated gene trees as described in Li et al. (2018). This null simulation accounts for variation in the number and percent of gene duplications associated with branch length and background birth/death rates among the sampled taxa. Significant bursts above this null indicate a deviation from the background birth and death rate as expected for episodic events like WGDs. We used Fisher's exact test to compare our observed MAPS results to the null simulations and identify significant episodes of duplication. All nodes are compared against the null model to identify significant episodes of gene duplication across a species tree. Once these significant episodes of gene duplication are identified, we used a second set of gene tree simulations to assess if they were consistent with a WGD. Again, we used Fisher's exact test to compare our observed numbers of duplications to the number of shared duplications expected with a WGD at a particular location in the phylogeny. If these increases in gene duplications were caused by a WGD, then we expect the numbers of shared gene duplications among extant taxa to be consistent with these positive simulations. By using these simulations and statistical methods, MAPS explicitly accounts for the number of duplications expected on branches of different lengths within species trees and provides a statistical test to assess if an episode of duplication is consistent with a potential ancient WGD.

It should be emphasized that we used a total evidence approach to infer WGDs in the 1KP capstone project. We combined evidence from single species Ks plots, pairwise ortholog divergence analyses, and multispecies MAPS analyses to identify ancient episodes of gene duplication consistent with WGDs and place them on our species tree. For example, we did not call a WGD based only on evidence from a MAPS analysis. In the few cases where the results of our different inference approaches conflicted, we relied on the weight of evidence from a majority of analyses and, if available, other analyses from the literature to infer a putative WGD. These were mostly cases where inferences from Ks plots, ortholog comparisons, and the previous literature agreed, but MAPS did not. In these cases, we recognized the event as a significant burst of gene duplication and indicated this in Supplemental text and tables, and labeled as blue squares on the ED WGD

Phylogeny Figure [8]. These events may be WGDs that should be analyzed in subsequent analyses with new data or methods.

3. Walk-through examples

To better demonstrate our approach for inferring ancient WGDs, we selected two examples from the 1KP analyses as walk-throughs. We chose the Pinaceae and Compositae ancient WGD analyses as examples (Fig. 4, 5) because these analyses represent different scales and complexities of duplication events. Previous analyses have found evidence for two rounds of WGD in the history of the Pinaceae [10], including a potential WGD in the ancestry of all seed plants [10,36]. However, other analyses have questioned the placement and/or existence of significant bursts of gene duplication in these lineages [37,38]. In contrast, the Compositae walk-through example has no conflict among studies, but is a complex nested paleohexaploidy in the ancestry of one of the largest families of flowering plants [39,40]. Inferring the location of the nested WGDs that comprise the paleohexaploidy, while also distinguishing other WGDs in these data, is a potentially challenging task for transcriptome based phylogenomic analyses. Below, we walk through our results for these examples and explain how we arrived at our inferences of a WGD (or not). It should be noted that we conducted a similar level of analysis and decision making process in the inference of all 244 putative WGDs in the 1KP capstone analysis.

Consistent with previous research [10,36], we observed evidence for at least two rounds of duplication in the ancestry of the Pinaceae. We observed two peaks of duplication consistent with two rounds of ancient WGDs in the history of three Pinaceae genera (*Pinus*, *Pseudotsuga*, and *Cedrus*; Fig. 4). Recent peaks of duplication in species of *Pinus*, *Pseudotsuga*, and *Cedrus* have a median Ks ~0.3 (Fig. 4a-c), older than their ortholog divergences (Ks ~ 0.18; Fig. 4c). These ortholog divergence analyses suggest the younger putative WGD in the three species is most likely shared by all Pinaceae. However, this putative WGD is not likely shared by other conifers because the ortholog divergences of the Pinaceae to other conifers is nearly twice the paralog divergence of the putative WGD. For example, ortholog divergences of members of the Pinaceae relative to members of the Cephalotaxaceae is Ks ~0.6 (Fig. 4c), consistent with this duplication event occurring after the divergence of these conifer families. The older peaks observed in *Pinus*, *Pseudotsuga*, and *Cedrus* have a median Ks ~1 (Fig. 4c), most likely shared by all seed plants but more recent than the divergence of seed plants and ferns (Ks ~3, estimated in 1KP capstone project).

As described above, we further assessed the nature of phylogenetic position of these putative WGDs using MAPS. We selected species of *Pinus*, *Pseudotsuga,* and *Cedrus* to represent Pinaceae in this MAPS analyses. We also selected species of *Araucaria* and *Ginkgo* to represent other gymnosperms, and species of *Equisetum* and *Selaginella* were used as outgroups. For the null simulations, we first simulated 3000 gene trees using the mean background gene duplication rate (λ) and gene loss rate (μ). We then randomly resampled 1000 trees without replacement from the total pool of gene trees 100 times to provide a measure of uncertainty of the percentage of subtrees at each node (Fig. 4d). At nodes corresponding to N1, N2, N4, and N5, we observed significantly more shared duplications than expected compared to the null simulations ($p < 0.01$) (Fig. 4d). For positive simulations, we incorporated a WGD at nodes N1, N2, N4, and N5 and simulated gene trees using the same methods described above. At the node representing the MRCA of Pinaceae (N2) and the node representing the MRCA of gymnosperms (N4), we identified an episodic burst of shared gene duplication that is statistically consistent with our positive simulations of WGDs (Fig. 4e). The results from our comparison to the null and positive simulations are consistent with those from Ks plots and ortholog divergence analyses described above, as well as those of our previous study in gymnosperms [10]. These results and another MAPS analysis in the 1KP capstone project (MAPS D1) show evidence consistent with a putative ancient WGD shared among all Pinaceae and another putative WGD that likely occurred in the ancestry of seed plants [8].

In addition to our analyses with the 1KP capstone dataset, other analyses have also inferred a putative WGD in the ancestry of all seed plants [10,36,37] and in the ancestry of different conifer families [10]. Consistent with our previous analyses [10], the relatively dense phylogenetic sampling of the 1KP allowed us to confirm that the putative seed plant WGD is not shared with monilophytes. A recent study proposed that cycads and *Ginkgo* might have shared another round of ancient WGD(s) [25]. However, other analyses in the 1KP capstone (MAPS D1 and related ortholog divergence analyses) using three species of cycads, *Ginkgo*, *Amborella*, and outgroups rejects this hypothesis. Instead, we find evidence that the signature detected by Roodt et al. (2017) in cycads and *Ginkgo* is most likely the putative seed plant WGD (One Thousand Plant Transcriptomes Initiative, 2019). In the 1KP and previous research [10], we also found evidence for other putative ancient WGDs in the ancestry of some families of conifers,

including the Pinaceae as described above. Using whole genome data from *Ginkgo biloba, Picea abies*, and *Pinus taeda*, a recent study does not find evidence in both Ks plots and phylogenomic analyses for the Pinaceae WGD [37]. The absence of a putative Pinaceae WGD peak in their Ks plot is possibly due to the quality of the genome assembly and annotation, or the scaling of their Ks plot which may obscure the peaks we observed in all Pinaceae taxa. In the 1KP capstone project, we consistently observed two peaks of gene duplication consistent with putative WGDs in all Ks plots from the 14 species of Pinaceae analyzed. Only one conifer species, *Picea abies*, was included in the analysis by Zwaenepoel and Van de Peer (2019). It is possible the lack of support for the Pinaceae WGD is due to the limited sampling of conifers, as they [37] demonstrated that taxon sampling can have a significant impact on WGD inference with taxon-dependent support for the well established eudicot hexaploidy [22,41–44]. Given the evidence from Ks plots, ortholog divergence, and MAPS analyses that we discussed above, our inference and placement of the putative Pinaceae and seed plants WGDs is currently the best explanation for these large scale gene duplication events. Future studies with new data, especially with higher quality gymnosperm genome assemblies, are needed to test these hypothesized WGDs.

To further demonstrate our total evidence approach to resolve complex ancient WGDs, we provide a walk-through of our analyses of ancient WGDs in the Asteraceae. We previously inferred two rounds of ancient WGD consistent with a paleohexaploidy in the ancestry of the Compositae [20,39,40]. The paleohexaploid nature of this WGD was later supported by synteny analyses of the sunflower and other Compositae genomes [45–47]. Given the great phylogenetic depth of sampling in the 1KP project and our introduction of a new statistical test for inferring WGD in MAPS [3] since our previous analysis, we re-evaluated the ancient WGDs with two new MAPS analyses and new data in the 1KP capstone (One Thousand Plant Transcriptomes Initiative, 2019). In one of the MAPS analyses (Fig. 5a), we selected species of *Cicerbita*, *Lactuca*, *Tragopogon*, *Leontopodium*, and *Carthamus* to represent the Compositae. Data from *Scaevola* and *Menyanthes* were used as outgroups. Our new analyses with the 1KP data confirmed the phylogenetic position of the paleohexaploidy in the ancestry of the Compositae (Fig. 5a). In the second analysis (Fig. 5b), we used the expanded phylogenetic sampling of the 1KP to more precisely locate an additional WGD in the ancestry of the Heliantheae previously inferred by Ks plots and ortholog divergence analyses [20] and synteny [46]. We selected

species of *Flaveria*, *Xanthium*, and *Helenium* to represent the tribe Heliantheae, and species of *Inula* and four other genera as outgroups. Our analysis of new 1KP data confirmed the location of the Heliantheae WGD with a significant peak of gene duplication consistent with a simulated WGD in the history of all Heliantheae sampled (Fig. 5). Our Compositae analyses in the 1KP allowed us to re-evaluate established WGDs using data from newly sampled taxa and more precisely locate these in the phylogeny. More than 100 of the 1KP WGDs were previously inferred and the expanded sampling of the 1KP dataset allowed us to more precisely place them as we did here in the Compositae.

4. Evaluation of WGD inferences

To evaluate our WGD inferences from the 1KP capstone project [8], we compared the consistency of our inferences with whole genome synteny analyses. Although limited in placing WGDs on a phylogeny because of the relatively low phylogenetic sampling of assembled genomes, synteny analysis using high quality genomes is generally considered the best approach for confirming an ancient WGD [43,48]. We compared the results of our Ks and MAPS analyses with analyses of WGDs from published synteny analyses of plant genomes (Fig. 6, SI_Table_1). Overall, we were able to make 65 comparisons of our Ks plot inferences and 43 comparisons of our MAPS phylogenomic analyses to syntenic analyses. Our inferences of WGDs with Ks plots and ortholog divergences were 100% consistent with syntenic analyses from either the same species or a close relative (Fig. 6, SI_Table_1). Despite a perception that Ks plots are difficult to interpret or unreliable, a recent study found that Ks plots analyses using best practices, as we did here, are highly robust [16]. Thus, the high consistency of our Ks plot inferences of WGDs with published genome analyses is not unexpected. We observed slightly lower consistency of our MAPS phylogenomic inferences of WGDs. Across the 43 synteny comparisons, we observed no false positives, but did observe six false negative results (Fig. 6, SI_Table_1). This tendency of our phylogenomic method towards false negatives and “missing” established WGDs is a known issue. There are cases of well established WGDs going undetected with different phylogenomic analyses, including *At-α* [49] and the eudicot gamma hexaploidy [37]. Although MAPS and other phylogenomic approaches are often viewed as more rigorous than single species approaches like Ks plots and synteny, these approaches are sensitive to a variety of parameters including gene tree sample size, taxon composition, gene tree occupancy, variation in branch lengths, variation in gene birth/death rates, and variation in gene retention and loss patterns

across the phylogeny, to name a few. Notably, we did not observe false positive inferences of WGDs with MAPS, and there does not appear to be reports of false positive inferences in the literature from other phylogenomic methods. However, false signals of large bursts of gene duplication, potentially on the scale consistent with a WGD, could be created by incomplete lineage sorting and quirks of gene tree reconciliation [32]. To minimize the potential biases of these types of phylogenomic methods in the 1KP capstone project, we aimed to use a total evidence approach that combined inferences across Ks plots, ortholog divergence analyses, and MAPS phylogenomic analyses to infer WGDs. Considering that we observed no false positives and high consistency of our Ks and MAPS analyses with syntenic results, we think our survey of WGDs across the phylogeny of green plants is reasonably robust and the combined approach minimized false positives. We expect that some of the 244 WGDs we inferred may move location or be merged as more data become available, and emphasize that the 138 newly inferred WGDs should be treated as hypotheses until confirmed with further data to corroborate the nature and precise timing of these large scale gene duplication events.

Availability of Supporting Data

https://bitbucket.org/barkerlab/1kp/src/master/

Funding

This research was supported by NSF grants IOS-1339156 and EF-1550838 to M.S.B.

References

1. Berthelot C, Brunet F, Chalopin D, Juanchich A, Bernard M, Noël B, et al. The rainbow trout genome provides novel insights into evolution after whole-genome duplication in vertebrates [Internet]. Nature Communications. 2014. Available from: http://dx.doi.org/10.1038/ncomms4657

2. Barker MS, Husband BC, Pires JC. Spreading Winge and flying high: The evolutionary importance of polyploidy after a century of study. Am J Bot. 2016;103:1139–45.

3. Li Z, Tiley GP, Galuska SR, Reardon CR, Kidder TI, Rundell RJ, et al. Multiple large-scale gene and genome duplications during the evolution of

hexapods. Proc Natl Acad Sci U S A. 2018;115:4713–8.

4. Wolfe KH, Shields DC. Molecular evidence for an ancient duplication of the entire yeast genome [Internet]. Nature. 1997. p. 708–13. Available from: http://dx.doi.org/10.1038/42711

5. Van de Peer Y, Maere S, Meyer A. The evolutionary significance of ancient genome duplications. Nat Rev Genet. 2009;10:725–32.

6. Mao Y, Satoh N. A Likely Ancient Genome Duplication in the Speciose Reef-Building Coral Genus, Acropora. iScience. 2019;13:20–32.

7. Wendel JF. The wondrous cycles of polyploidy in plants. Am J Bot. 2015;102:1753–6.

8. One Thousand Plant Transcriptomes Initiative. A phylogenomic view of evolutionary complexity across green plants. Nature.

9. Barker MS, Dlugosch KM, Dinh L, Challa RS, Kane NC, King MG, et al. EvoPipes.net: Bioinformatic Tools for Ecological and Evolutionary Genomics. Evol Bioinform Online. ncbi.nlm.nih.gov; 2010;6:143–9.

10. Li Z, Baniaga AE, Sessa EB, Scascitelli M, Graham SW, Rieseberg LH, et al. Early genome duplications in conifers and other seed plants. Sci Adv. American Association for the Advancement of Science; 2015;1:e1501084.

11. Zhang Z, Schwartz S, Wagner L, Miller W. A greedy algorithm for aligning DNA sequences. J Comput Biol. 2000;7:203–14.

12. Ma B, Tromp J, Li M. PatternHunter: faster and more sensitive homology search. Bioinformatics. 2002;18:440–5.

13. Birney E, Clamp M, Durbin R. GeneWise and Genomewise. Genome Res. 2004;14:988–95.

14. Goodstein DM, Shu S, Howson R, Neupane R, Hayes RD, Fazo J, et al. Phytozome: a comparative platform for green plant genomics. Nucleic Acids Res. 2012;40:D1178–86.

15. Yang Z. PAML 4: phylogenetic analysis by maximum likelihood. Mol Biol Evol. 2007;24:1586–91.

16. Tiley GP, Barker MS, Burleigh JG. Assessing the performance of Ks plots for detecting ancient whole genome duplications. Genome Biol Evol [Internet]. 2018; Available from: http://dx.doi.org/10.1093/gbe/evy200

17. Vanneste K, Van de Peer Y, Maere S. Inference of genome duplications from age distributions revisited. Mol Biol Evol. SMBE; 2013;30:177–90.

18. Cui L, Wall PK, Leebens-Mack JH, Lindsay BG, Soltis DE, Doyle JJ, et al. Widespread genome duplications throughout the history of flowering plants. Genome Res. genome.cshlp.org; 2006;16:738–49.

19. Benaglia T, Chauveau D, Hunter D, Young D. mixtools: An R Package for Analyzing Mixture Models. Journal of Statistical Software, Articles. 2009;32:1–29.

20. Barker MS, Kane NC, Matvienko M, Kozik A, Michelmore RW, Knapp SJ, et al. Multiple paleopolyploidizations during the evolution of the Compositae reveal parallel patterns of duplicate gene retention after millions of years. Mol Biol Evol. 2008;25:2445–55.

21. Shi T, Huang H, Barker MS. Ancient genome duplications during the evolution of kiwifruit (Actinidia) and related Ericales. Ann Bot. 2010;106:497–504.

22. Barker MS, Vogel H, Schranz ME. Paleopolyploidy in the Brassicales: analyses of the Cleome transcriptome elucidate the history of genome duplications in Arabidopsis and other Brassicales. Genome Biol Evol. 2009;1:391–9.

23. Cannon SB, McKain MR, Harkess A, Nelson MN, Dash S, Deyholos MK, et al. Multiple polyploidy events in the early radiation of nodulating and nonnodulating legumes. Mol Biol Evol. 2015;32:193–210.

24. Yang Y, Moore MJ, Brockington SF, Soltis DE, Wong GK-S, Carpenter EJ, et al. Dissecting Molecular Evolution in the Highly Diverse Plant Clade Caryophyllales Using Transcriptome Sequencing. Mol Biol Evol. 2015;32:2001–14.

25. Roodt D, Lohaus R, Sterck L, Swanepoel RL, Van de Peer Y, Mizrachi E. Evidence for an ancient whole genome duplication in the cycad lineage. PLoS One. 2017;12:e0184454.

26. Smith SA, Brown JW, Yang Y, Bruenn R, Drummond CP, Brockington SF, et al. Disparity, diversity, and duplications in the Caryophyllales. New Phytol. 2018;217:836–54.

27. Emms DM, Kelly S. OrthoFinder: solving fundamental biases in whole genome comparisons dramatically improves orthogroup inference accuracy. Genome Biol. 2015;16:157.

28. Mirarab S, Nguyen N, Warnow T. PASTA: Ultra-Large Multiple Sequence Alignment. In: Sharan R, editor. Research in Computational Molecular Biology. Cham: Springer International Publishing; 2014. p. 177–91.

29. Katoh K, Misawa K, Kuma K-I, Miyata T. MAFFT: a novel method for rapid multiple sequence alignment based on fast Fourier transform. Nucleic Acids Res. 2002;30:3059–66.

30. Edgar RC. MUSCLE: multiple sequence alignment with high accuracy and high throughput. Nucleic Acids Res. 2004;32:1792–7.

31. Stamatakis A. RAxML version 8: a tool for phylogenetic analysis and post-analysis of large phylogenies. Bioinformatics. 2014;30:1312–3.

32. Hahn MW. Bias in phylogenetic tree reconciliation methods: implications for vertebrate genome evolution. Genome Biol. 2007;8:R141.

33. Smith SA, Moore MJ, Brown JW, Yang Y. Analysis of phylogenomic datasets reveals conflict, concordance, and gene duplications with examples from animals and plants. BMC Evol Biol. 2015;15:150.

34. Rabier C-E, Ta T, Ané C. Detecting and locating whole genome duplications on a phylogeny: a probabilistic approach. Mol Biol Evol. 2014;31:750–62.

35. Sjöstrand J, Arvestad L, Lagergren J, Sennblad B. GenPhyloData: realistic simulation of gene family evolution. BMC Bioinformatics. 2013;14:209.

36. Jiao Y, Wickett NJ, Ayyampalayam S, Chanderbali AS, Landherr L, Ralph PE, et al. Ancestral polyploidy in seed plants and angiosperms. Nature. 2011;473:97–100.

37. Zwaenepoel A, Van de Peer Y. Inference of Ancient Whole-Genome Duplications and the Evolution of Gene Duplication and Loss Rates. Mol Biol Evol. 2019;36:1384–404.

38. Ruprecht C, Lohaus R, Vanneste K, Mutwil M, Nikoloski Z, Van de Peer Y, et al. Revisiting ancestral polyploidy in plants. Sci Adv. 2017;3:e1603195.

39. Barker MS, Li Z, Kidder TI, Reardon CR. Most Compositae (Asteraceae) are descendants of a paleohexaploid and all share a paleotetraploid ancestor with the Calyceraceae. American Journal of [Internet]. Wiley Online Library; 2016; Available from: https://onlinelibrary.wiley.com/doi/abs/10.3732/ajb.1600113

40. Huang C-H, Zhang C, Liu M, Hu Y, Gao T, Qi J, et al. Multiple Polyploidization Events across Asteraceae with Two Nested Events in the Early History Revealed by Nuclear Phylogenomics. Mol Biol Evol. SMBE; 2016;33:2820–35.

41. Jiao Y, Leebens-Mack J, Ayyampalayam S, Bowers JE, McKain MR, McNeal J, et al. A genome triplication associated with early diversification of the core

eudicots. Genome Biol. 2012;13:R3.

42. Jaillon O, Aury J-M, Noel B, Policriti A, Clepet C, Casagrande A, et al. The grapevine genome sequence suggests ancestral hexaploidization in major angiosperm phyla. Nature. 2007;449:463–7.

43. Lyons E, Pedersen B, Kane J, Alam M, Ming R, Tang H, et al. Finding and comparing syntenic regions among Arabidopsis and the outgroups papaya, poplar, and grape: CoGe with rosids. Plant Physiol. 2008;148:1772–81.

44. Vekemans D, Proost S, Vanneste K, Coenen H, Viaene T, Ruelens P, et al. Gamma paleohexaploidy in the stem lineage of core eudicots: significance for MADS-box gene and species diversification. Mol Biol Evol. 2012;29:3793–806.

45. Reyes-Chin-Wo S, Wang Z, Yang X, Kozik A, Arikit S, Song C, et al. Genome assembly with in vitro proximity ligation data and whole-genome triplication in lettuce. Nat Commun. 2017;8:14953.

46. Badouin H, Gouzy J, Grassa CJ, Murat F, Staton SE, Cottret L, et al. The sunflower genome provides insights into oil metabolism, flowering and Asterid evolution. Nature. 2017;546:148–52.

47. Song C, Liu Y, Song A, Dong G, Zhao H, Sun W, et al. The Chrysanthemum nankingense Genome Provides Insights into the Evolution and Diversification of Chrysanthemum Flowers and Medicinal Traits [Internet]. Molecular Plant. 2018. p. 1482–91. Available from: http://dx.doi.org/10.1016/j.molp.2018.10.003

48. Tang H, Bowers JE, Wang X, Ming R, Alam M, Paterson AH. Synteny and Collinearity in Plant Genomes [Internet]. Science. 2008. p. 486–8. Available from: http://dx.doi.org/10.1126/science.1153917

49. Tiley GP, Ané C, Burleigh JG. Evaluating and Characterizing Ancient Whole-Genome Duplications in Plants with Gene Count Data. Genome Biol Evol. 2016;8:1023–37.

Fig. 1 Histograms of the age distribution of gene duplications (Ks plots) with mixture models of inferred WGDs for **(a)** *Pandorina morum* (green algae), no inferred WGD peak. **(b)** *Sphagnum recurvatum* (Moss), inferred WGD peak median Ks=0.38. **(c)** *Diphasiastrum digitatum* (Lycophyte), inferred WGD peaks median Ks=0.42, 1.62. **(d)** *Ceratopteris thalictroides* (Fern), inferred WGD peak median Ks=1.08. **(e)** *Pseudotsuga wilsoniana*

(Gymnosperm), inferred WGD peak median Ks=0.38, 1.18. **(f)** *Ipomoea nil* (Angiosperm) inferred WGD peak median Ks=0.66. Histogram x-axis scale is Ks 0–2. The mixture model distributions consistent with inferred ancient WGDs are highlighted in yellow.

Fig. 2 Histograms of the age distribution of gene duplications (Ks plots) with mixture models of inferred WGDs for **(a)** *Pandorina morum* (green algae), no inferred WGD peak. **(b)** *Sphagnum recurvatum* (Moss), inferred WGD peak median Ks=0.38. **(c)** *Diphasiastrum digitatum* (Lycophyte), inferred WGD peaks median Ks=0.42, 1.62. **(d)** *Ceratopteris thalictroides* (Fern), inferred WGD peak median Ks=1.08, 3.07. **(e)** *Pseudotsuga wilsoniana* (Gymnosperm), inferred WGD peak median Ks=0.38, 1.18. **(f)** *Ipomoea nil* (Angiosperm) inferred WGD peak median Ks=0.66, 2.15. Histogram x-axis scale is Ks 0–5. The mixture model distributions consistent with inferred ancient WGDs are highlighted in green.

Fig. 3 Increasing taxon occupancy decreases the inflation of mapped duplications towards the root of the species tree in MAPS. The black line represents the MAPS result without the minimum taxon requirement. The blue line represents the MAPS results with a 35% minimum taxa requirement. The red line represents the MAPS results with a 45% minimum taxa requirement. N1 corresponds to the tip node, and the last node (eg: N4 in **(a)**) corresponds to the root node. * represents nodes associated with inferred WGDs. **(a)** 1KP MAPS result of eudicot ancient hexaploidy event, N2 represents the node associated with this paleohexaploidy event. See MAPS E21 in the One Thousand Plant Transcriptomes Initiative, 2019 for details. **(b)** N2 represents the node associated with an inferred Pinaceae WGD, N4 represents the node associated with the inferred seed plant WGD. See Fig. 4c, d for the phylogeny. **(c)** N4 represents node associated with the paleohexaploidy event shared by most Compositae, see Fig. 5a for the phylogeny. **(d)** N4 represents node associated with the Heliantheae ancient WGD, see Fig. 5b for the phylogeny.

Fig. 4 Histograms of the age distribution of gene duplications (Ks plots), ortholog divergences, and MAPS results for the Pinaceae ancient WGD. **(a)** and **(b)** Histograms of the age distribution of gene duplications (Ks plots) with mixture models of inferred WGDs for *Pseudotsuga wilsoniana* (Gymnosperm), inferred WGD peak median Ks=0.38, 95% CI (0.371, 0.386) and 1.18, 95% CI (1.163, 1.195). **(a)** Histogram x-axis scale is Ks 0–2. The mixture model distributions consistent with inferred ancient WGDs are

highlighted in yellow. **(b)** Histogram x-axis scale is Ks 0–5. The mixture model distributions consistent with inferred ancient WGDs are highlighted in green. **(c)** Combined Ks plot of the gene age distributions of *P. wilsoniana* (blue), *Pinus radiata* (black), *Cedrus libani* (gray), and ortholog divergences of *Pinus* vs. *Cedrus* (orange) and *Cedrus* (Pinaceae) vs. *Cephalotaxus* (Cephalotaxaceae) (red). The median peaks for these plots are highlighted. *Pinus radiata* (black), inferred WGD peak medians at Ks=0.37, 95% CI (0.365, 0.380) and 1.16, 95% CI (1.142, 1.172). *Cedrus libani* (gray), inferred WGD peak medians at Ks=0.33, 95% CI (0.316, 0.336) and 1.08, 95% CI (1.061, 1.099). **(d)** and **(e)** MAPS results from observed data, null and positive simulations on the associated phylogeny. **(d)** Percentage of subtrees that contain a gene duplication shared by descendant species at each node, results from observed data (red line), 100 resampled sets of null simulations (multiple black lines). **(e)** Percentage of subtrees that contain a gene duplication shared by descendant species at each node, results from observed data (red line), and positive simulations (multiple gray lines). The orange oval corresponds to the location of an inferred WGD in Pinaceae. The green oval corresponds to the location of an inferred WGD in seed plants.

Fig. 5 Asteraceae MAPS results from observed data, null, and positive simulations on the associated phylogeny. **(a)** Percentage of subtrees that contain a gene duplication shared by descendant species at each node, results from observed data (red line), 100 resampled sets of null simulations (multiple black lines) and positive simulations (multiple gray lines). The red oval corresponds to the paleohexaploidy event in the Compositae. **(b)** Percentage of subtrees that contain a gene duplication shared by descendant species at each node, results from observed data (red line), 100 resampled sets of null simulations (multiple black lines) and positive simulations (multiple gray lines). The blue oval corresponds to the Heliantheae ancient WGD.

Fig. 6 Consistency of the 1KP Ks and MAPS inferences of WGD with results from published synteny analyses of plant genomes. Consistent results represented by blue and false negative results represented by red. There were no false positives in our inferences of WGDs compared to those from published synteny analyses.

Figure

Click here to access/download;Figure;Fig_1.pdf

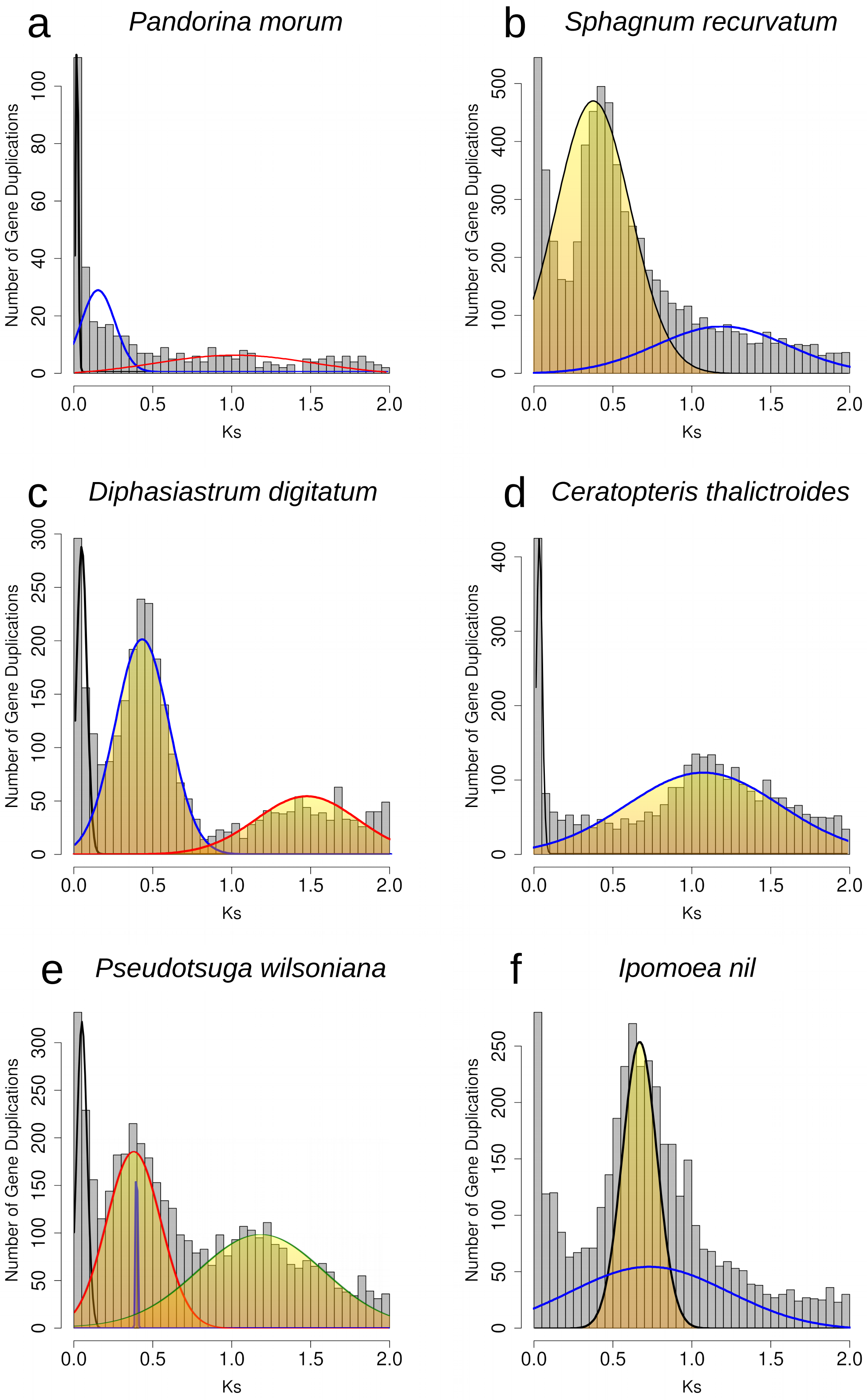

Figure

Click here to access/download;Figure;Fig_2.pdf

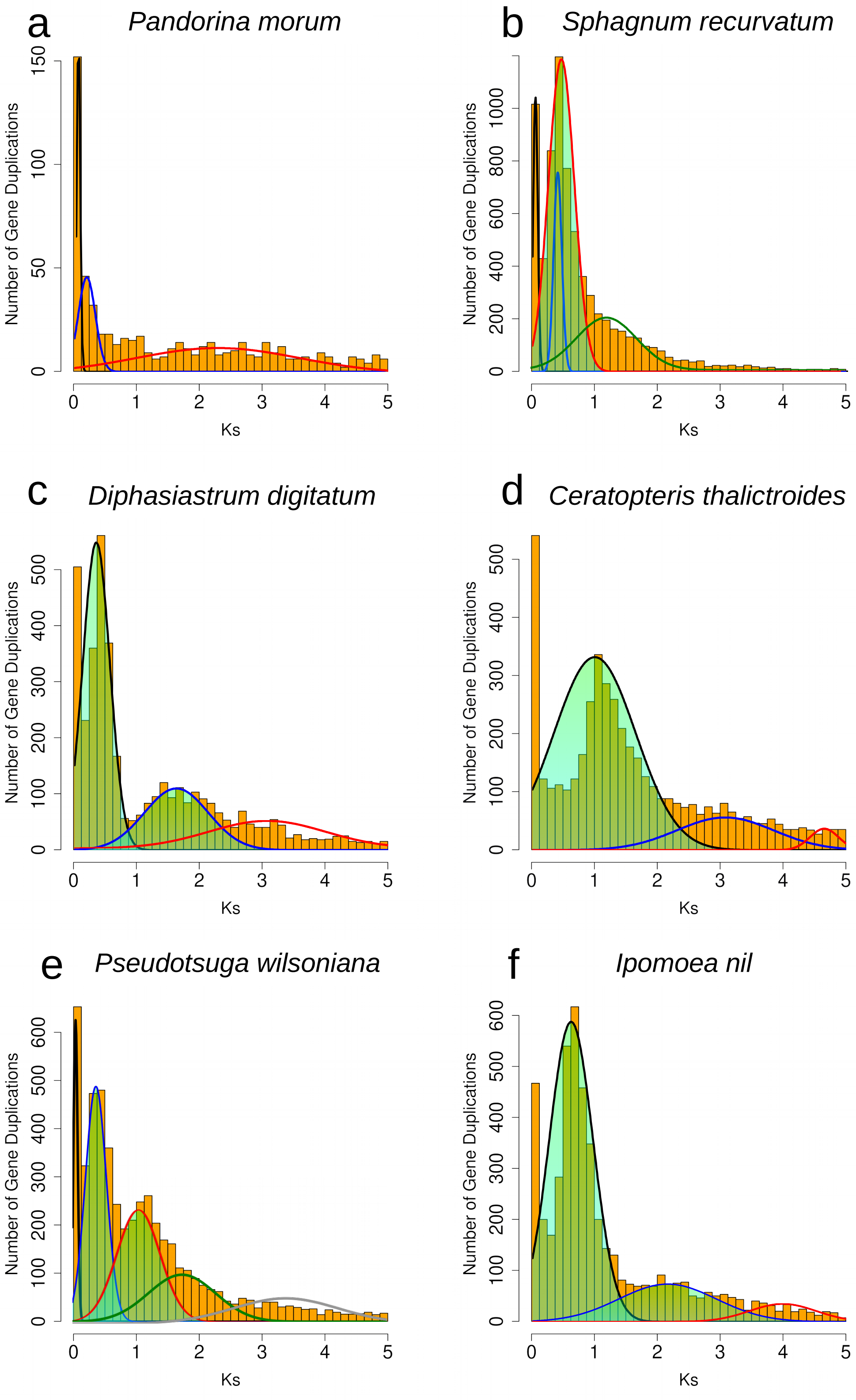

Click here to access/download;Figure;Fig_3.pdf

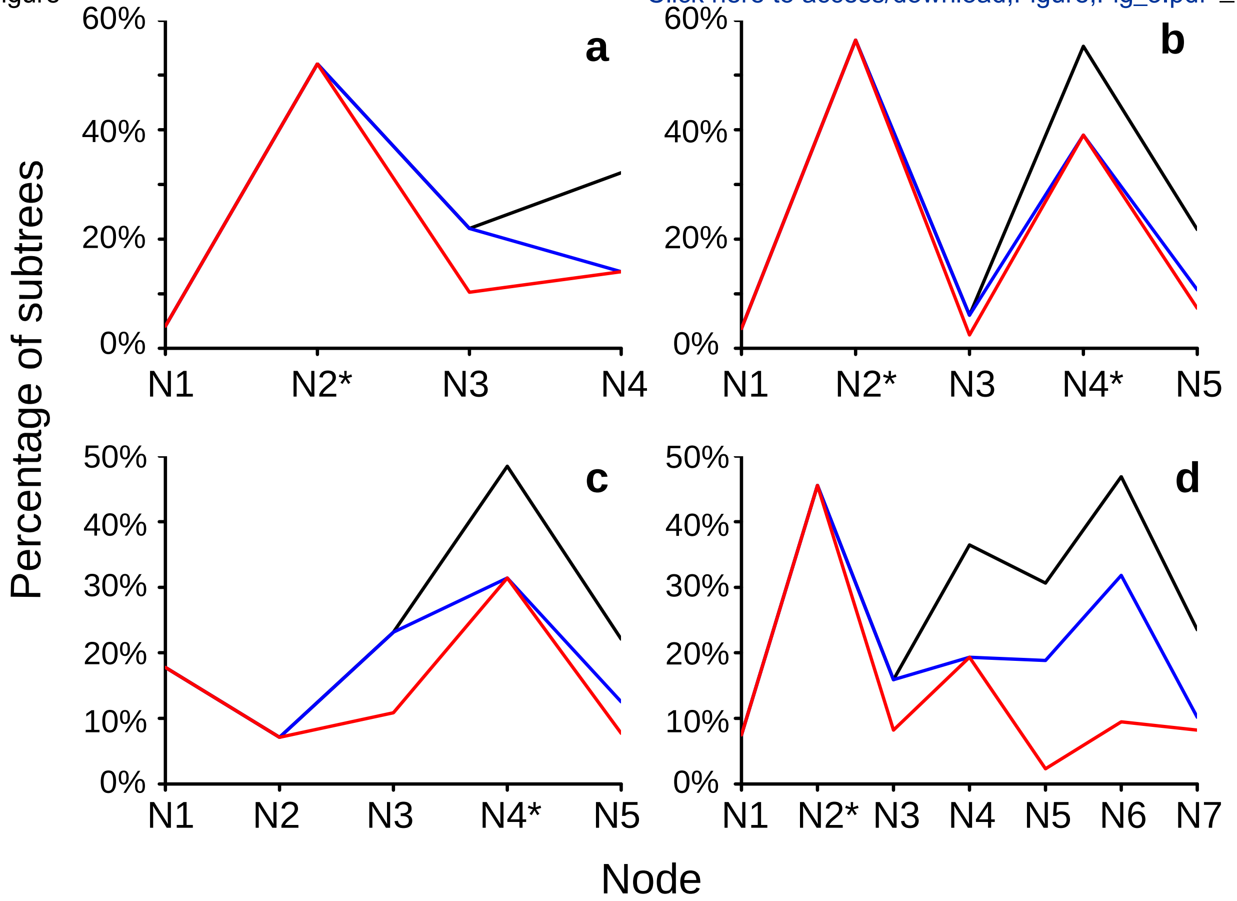

Figure

Click here to access/download;Figure;Fig_4.pdf

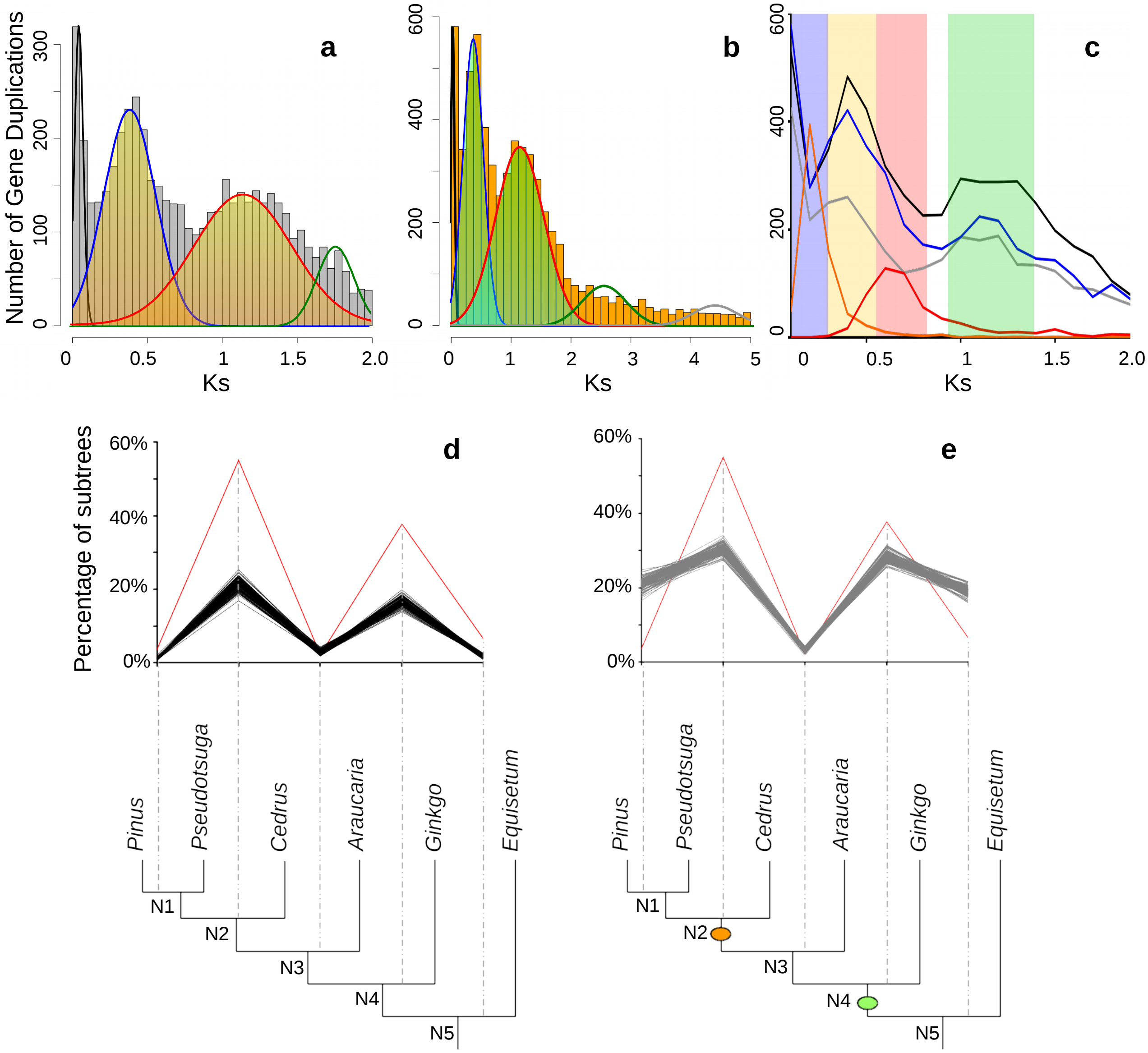

Click here to access/download;Figure;Fig_5.png

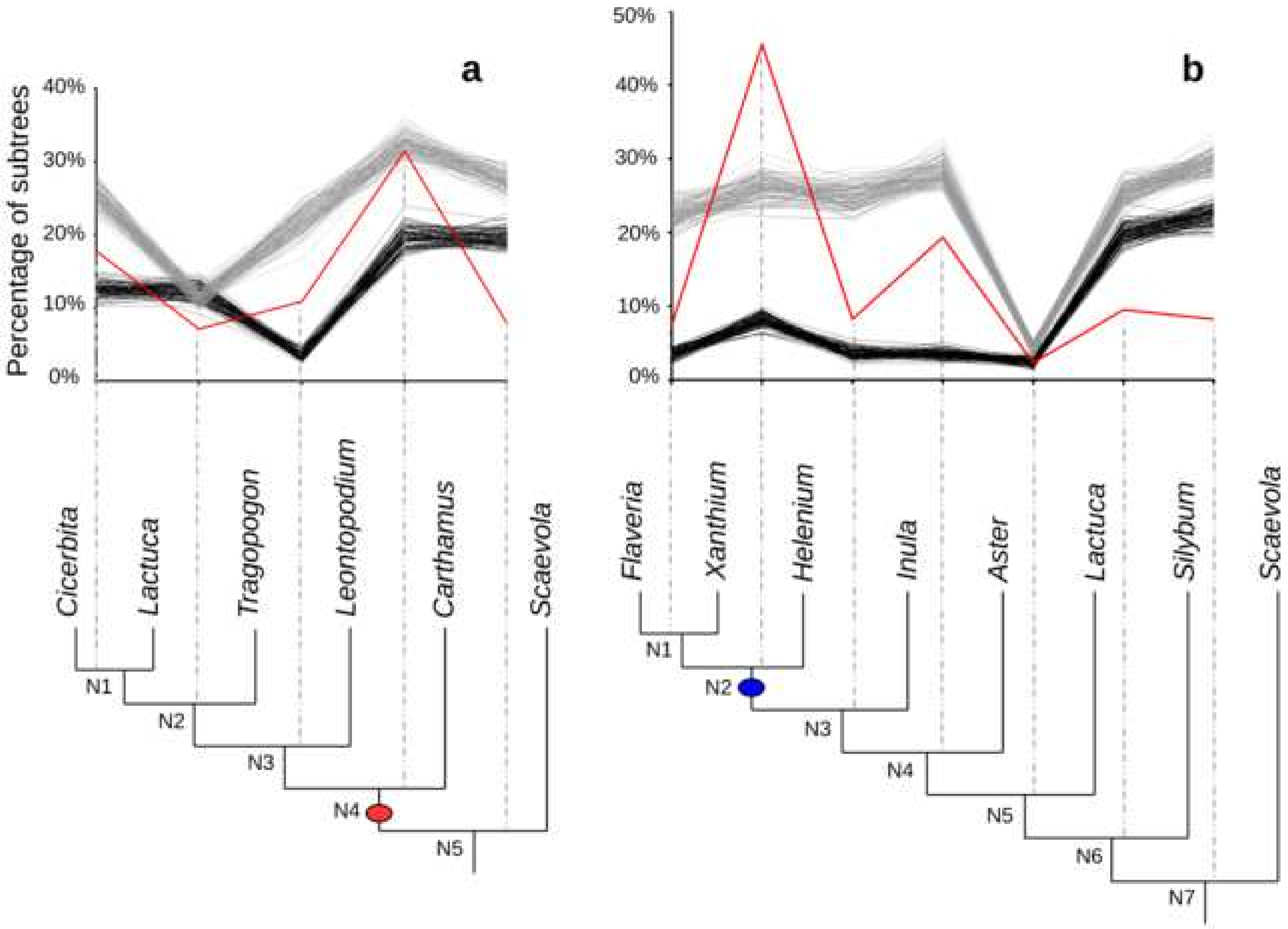

Click here to access/download;Figure;Fig_6.png

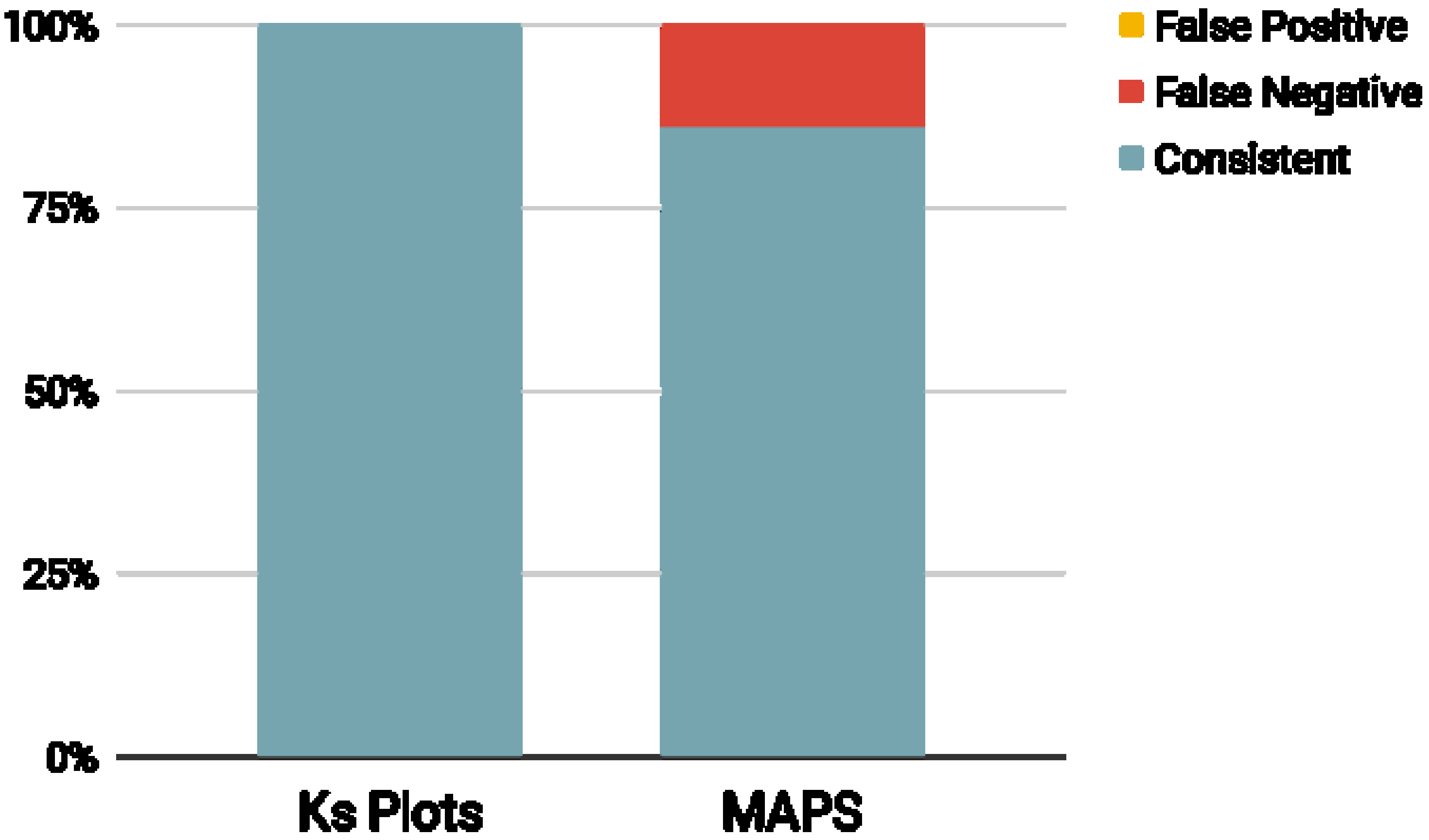

Click here to access/download
Supplementary Material
SI_Table_1_WGD_synteny_survey.xlsx
