# Supplementary material for: Inferring putative ancient whole-genome duplications in the 1000 Plants (1KP) initiative: access to gene family phylogenies and age distributions
Source: Gigascience. 2020 Feb 11;9(2):giaa004. doi: 10.1093/gigascience/giaa004 (PMC7011446; doi:10.1093/gigascience/giaa004)
Supplement: giaa004_Response_to_Reviewer_Comments_Original_Submission [file giaa004_response_to_reviewer_comments_original_submission.pdf]

Author's Response To Reviewer Comments

Close

Dear Scott,

We thank you and both reviewers for the thorough reviews and suggestions to improve our manuscript. We have made a number of revisions to the manuscript to address the specific concerns of reviewers to better demonstrate the goal of this accompaniment paper. These included revising the abstract and adding a new Context section. We also revised and included detailed methodology for the DupPipe pipeline. We feel the suggested revisions have improved the quality of the manuscript and we thank the reviewer #1 for bring up many valid suggestions in our analyses. Overall, our revised manuscript is clearer on demonstrate the value of this accompaniment. Below, we respond to each reviewer comment and describe in detail how we have addressed the issues identified and the corresponding revisions to our manuscript. Our responses are typed here and revisions to the manuscript itself are highlighted in yellow.

Zheng Li

Reviewer reports:

Reviewer #1: Whole-genome duplications (WGDs) play an important role in the evolution of plants and animals. Investigating the WGDs in plants with 1KP genomic data provides us a big picture of genome evolution in green plants. Although lots of publications have addressed this question, Li's manuscript provides a systematical analysis for the 1KP dataset and then it opens a road for further WGDs' research in the future. Therefore, I recommend this manuscript to publish in the journal.
We greatly appreciated reviewer #1's suggestions and comments. Please see our detailed response below.

However, I have a few comments on the analysis and the manuscript's structure.

Major comments:

1. What is the main point in this manuscript? "Building a total evidence approach for WGDs' research" or "Investigating WGDs in green plants with 1KP dataset using a total evidence approach".
This manuscript is one of the accompaniment papers to the 1KP Nature capstone paper. All these accompaniment papers aim to provide detailed methodology, validation, and provide a guide to describe the complex data of the accompanying Nature article. In this accompaniment manuscript, we aim to provide provide expanded methodology of the WGD analyses by using walkthrough examples, provide validation to our ancient WGD inferences, and release relevant data and other materials from the 1KP ancient WGD analyses.

The abstract makes me feel that this manuscript's main point on "Investigating WGDs in green plants with 1KP dataset using a total evidence approach", yet the main text makes me feel that the manuscript describes "a total evidence approach" for WGDs' research and using 10KP as a system.
We appreciate the reviewer's comment on the abstract. This is a very good point. We have revised our abstract and added a new Context section. We hope this makes more clear for the goal of this accompaniment paper.

For my understanding, "a total evidence approach" has been applied and described in Jiao's paper and Mao's previous paper (Jiao, Yuannian, et al; Nature; 2011; Mao, Yafei, et al; iScience; 2019). I recommend authors to re-organize the main text to show the point that "Investigating WGDs in green plants with 1KP dataset using a total evidence approach" with INTRODUCTION, METHODS, RESULTS, AND DISCUSSION. This would make this manuscript clear and have much impact on plant WGDs' research in the future.
As we described the purpose of this manuscript above, this is accompaniment papers to the 1KP Nature

capstone paper. The format suggested by the reviewer is for a Research Article. In this manuscript, we follow the standard and format for a Data Note in GigaScience.

Plus, a personal taste, I would like to use another word to replace "a total evidence approach", what is the "total evidence"? We might have other methods to infer WGDs and do better in the future. (if authors would like to use "a total evidence approach" in the revised manuscript, I have no problem with it).
We appreciate the reviewer's comment on this part. As we discussed in this manuscript, we agree with the reviewer that future improvement in methods and data will better infer ancient WGDs across the tree of life. For the wording of 'a total evidence approach', this term is commonly used in the field of Phylogenetics and Systematics (For example: Ronquist et al. 2012 Syst. Bio.; Arrigo et al. 2013 AJB; Gomes-Silva et al. 2017 Cladistics). It describes the philosophical principle that we will evaluate use multiple lines of evidence to test a hypothesis. In Systematics for example, a total evidence approach requires one to bring in molecular, morphological, and as many data as possible to understand the evolutionary history of organisms. In the 1KP Nature capstone analysis, we use the total evidence approach to infer ancient WGDs by using gene age distribution analysis from transcriptome of single species, orthologous divergence estimation between species to place WGDs, MAPS phylogenomic analyses from transcriptomes of multiple species to infer and place WGDs, and syntenic evidence from published plant genomes to compare with our inferences. We believe it is appropriate to call combining these different lines of evidence as 'a total evidence approach' for the 1KP Nature capstone analysis.

2. In abstract, "we release 383,679 nuclear gene family phylogenies and 2,306 gene age distribution (Ks) plots from the 1KP capstone paper." Could you release original data rather plots?
Yes, we added the original output files into the bitbucket repository.

In the method 1.1 "DupPipe analyses of WGDs from transcriptomes of single species", could you release the all orthogroups data which were generated by DupPipe pipeline? As well, in the method 1.3 "MAPS analyses of WGDs from transcriptomes of multiple species", could you release the orthogroups data which were generated by OrthoFinder?
The orthogroups data are available in the 1KP bitbucket repository.
https://bitbucket.org/barkerlab/1kp/src/master/
For the DupPipe pipeline, all gene family output can now be found in the original output files. For MAPS, all the orthogroups data that are used in MAPS which were generated by OrthoFinder can be found in the tar files in the 1KP bitbucket repository. These tar files are organized by each MAPS analysis. For each gene family, an alignment file and a gene family phylogeny in newick format can be downloaded from the bitbucket repository.

Curious, why do authors use two different methods (DupPipe and OrthoFinder) to generate orthogroups for Ks and MAPS analysis, respectively? (I do not think the methods will have a big impact on the final results, but I'd like to know more about the authors' experimental design.)
The DupPipe pipeline uses reciprocal best hit followed by single linkage clustering to generate gene families and then uses PAML to calculate the node Ks. Using the reciprocal best hit approach to construct gene families from a single transcriptome or genome is commonly used and highly efficient (Tatusov et al. 1997 Science, Bork et al. 1998 J. Mol. Biol.). As we demonstrated recently (Tiley et al. 2018 GBE) and discussed previously (Barker et al. 2008 MBE), using the node Ks values from gene families rather than the pairwise Ks value reduces error in estimating the Ks values of duplication events and improves the resolution of Ks peaks (Tiley et al. 2018 GBE). The DupPipe analyses are focussed on single species. In MAPS, we search for ancient WGDs across genomic data from multiple species. These species might be highly diverged, for example, mosses and seed plants diverged 600 Mya. Given that orthogroups need to be clustered in a much larger dataset (multiple transcriptomes or genomes from many species) and sequences that may be highly divergent, we use a more sophisticated gene family clustering algorithm such as OrthoMCL (Li et al, 2003) or OrthoFinder (Emms et al. 2015) to address these challenges.

3. I am not an expert for statistical tests, I have no more comments on statistical test in this manuscript. All look good for me by far.
We appreciate the reviewer's comment on our statistical tests.

4. In method 1.3, authors used RAxML to reconstruct gene trees, and then I am wondering whether authors used Partitionfinder to select best-fit partitioning schemes as input for RAxML to avoid gene tree bias. If so, authors should mention this in the method section. If not, I recommend the authors to perform this analysis. Alternatively, authors could use IQ-TREE to perform best-fit partitioning schemes

finding and tree reconstruction together automatically. My program TREEasy (https://github.com/MaoYafei/TREEasy) might be helpful for a large amount of tree reconstruction with IQ-TREE. However, authors should choose the easier way for themselves to solve this problem, no need to use TREEasy as a must.
We used RAxML for constructing gene trees because it is implemented in PASTA, and used 'PROTWAGCAT' for AA model which is the default setting in PASTA. Partitionfinder is written before the data explosion in phylogenomics (Lanfear et al, 2012 MBE). It is considered to be too slow for large genomic and transcriptomic datasets (Lanfear et al. 2017 MBE). We will consider using Partitionfinder 2 (Lanfear et al. 2017 MBE) or TREEasy in the future.

5. About Figures, I recommend authors to consider "integrate" Figure 1 and Figure 2 together. If so, it would be more concise for readers. Moreover, If I were authors, I would like to use a table to present Figure 6 (you already have it in your supplementary file).
We thank the reviewer's comment on the Figures. For Figure 1 and Figure 2, we believe it is more appropriate to have two different figure panels. As we stated in text "we plotted and presented two sets of histograms with x-axis scales of Ks = 2 and Ks = 5 to assess WGDs at different resolutions (Fig. 1, Fig. 2)". We also posted these two different sets of histograms on the bitbucket repository. It is clearer to show these are two different sets of histograms by having two separate figures. It also makes each plot bigger. This helps visualizing the details of the Ks plots. For Figure 6, the current figure provides a simple and clear visualization of the consistent, false positive, and false negative rates. Changing Figure 6 into a table will likely lose the simple visualization and make it harder to process the same information. For readers that are interested in any details of this validation, one can look up or search related information in the SI Table 1.

6. About re-organization of the manuscript as I suggested above, if authors would like to do it, I also suggest that authors could mention WGDs in other clades rather than plants. This might attract more readers who are not studying WGDs in plants.
We have added this at the beginning of the context section and cited relevant papers.

Minor comments:

1. 1KP_WGD_phylogeny.pdf in (https://bitbucket.org/barkerlab/1kp/src/master/1KP_WGD_phylogeny.pdf) might be truncated. See the top of PDF, some lineages did not show in the pdf file.
We checked the pdf file. This file is not truncated for us on at least two different computers.

2. In "Walk-through examples", like "a median Ks ~0.3". Could you also put the 95% CI there? Also, could you put the line number in the revised manuscript? Sometimes, it is really hard to point out sentences.
We thank the reviewer's comment. For "a median Ks ~0.3", we want to state that both Pinus, Pseudotsuga, and Cedrus has a WGD peak with a median Ks around 0.3. This pattern can be easily observed in Fig. 4c. We also highlighted it in yellow in Fig. 4c. We added the median Ks value estimated from the mixture model and 95% CI for both Pinus, Pseudotsuga, and Cedrus as requested in the Fig. 4c legend. All median Ks values for WGD peaks inferred by mixture models are also provided in the 1KP capstone paper as a searchable spreadsheet.

We add the version of the revised manuscript with line numbers.

3. In "This tendency of our phylogenomic method towards false negatives and "missing" established WGDs is a known issue. There are cases of well-established WGDs going undetected with different phylogenomic analyses, including At-[43]". "AT-[]" might have some formatting error here.
This is a format converting error. We corrected it to 'At-α'.

4. In "In this project, we used the approach described in Tiley et al. 2018. Previous analyses". Is "in this study" better than "In this project"?
We change 'in this project' to 'in the 1KP capstone project' to avoid any confusion.

5. Tiley et al. 2018 paper also mentioned that inferred WGDs are more robust when Ks<1 rather than Ks<2. But, I think Ks<2 is acceptable. Maybe it is worth to mention this in the discussion.
We agree with the reviewer. We stated in the manuscript that there is reasonable power to infer ancient WGDs when Ks < 2 in this manuscript, please see line 87-98. We also cited Vanneste et al. 2013 and

Tiley et al. 2018 which discussed this issue in depth.

6. Using transcriptomes to infer WGD with age distribution sometime is dangerous because transcriptomes assembly mistakes or incomplete transcriptomes assembly would lead to loss of WGD signals. Authors should mention this in the discussion.
We mentioned this in the Evaluation of WGD inferences section. As we stated in line 377-379 'Despite a perception that Ks plots are difficult to interpret or unreliable, a recent study found that Ks plots analyses using best practices, as we did here, are highly robust'. Here, our evaluation of Ks plot WGD inferences were 100% consistent with published genome analyses. Stating transcriptomes assembly mistakes or incomplete transcriptomes assembly would lead to loss of WGD signals is also misleading. Potential issues or mistakes in assembly can also apply to whole genome sequencing.

Reviewer #2: In this manuscript, the authors showed the detailed information about the 1KP data and the pipline they used in the WGD inference. As it is a companion paper, I think it is very necessary and useful to have such a description for the data anslysis.

However, I found that the explanation of the pipline is not very clear for the DupPipe analyses. The authors should have a more detailed description, especially for readers who are not familar with the DupPipe pipeline.
We greatly appreciate reviewer #2 comments and suggestions. We revised the section for the DupPipe analyses by adding methodological details. Please also be aware that DupPipe is a published pipeline and we cited the original papers that describe the pipeline in more detail (Barker et al. 2008 MBE and Barker et al. 2010 Evo. Bioinfo.)

such as:

How they construct gene families using the transcriptomes from only one species?
The DupPipe pipeline uses a reciprocal best blast hit and single linkage clustering to construct gene families from a single genome or transcriptome. Using the reciprocal best hit approach to construct gene families from a single transcriptome or genome is commonly used and highly efficient (Tatusov et al. 1997 Science, Bork et al. 1998 J. Mol. Biol.). We analyzed these gene family clusters with PAML to calculate the node Ks rather than the pairwise Ks value (Barker et al. 2010 Evo. Bioinfo.). This approach can reduce error in estimating Ks values of duplication events and improve the resolution of Ks peaks (Tiley et al. 2018 GBE). We have added the details for gene family clustering in the DupPipe section. Please see line 74-82. Please notice that DupPipe is a published pipeline, and we cited the original papers that describe the pipeline in more detail (Barker et al. 2008 MBE and Barker et al. 2010 Evo. Bioinfo.)

and How they identify peaks in the distribution of KS values?
The second paragraph of the section '2.1 DupPipe analyses of WGDs from transcriptomes of single species' describes how we identify significant features in the gene age distributions that may correspond to WGDs. We used two statistical tests: Kolmogorov–Smirnov goodness of fit tests and mixture models. 1) By using the using a K–S goodness of fit test, we first identified taxa with potential WGDs by comparing their paralog ages to a simulated null distribution without ancient WGDs. 2) For taxa with evidence for a significant peak relative to the null, we used a mixture model implemented in the mixtools R package to identify significant peaks of gene duplication consistent with WGDs. Please see line 104-116.

Close
