# Supplementary material for: Inferring putative ancient whole-genome duplications in the 1000 Plants (1KP) initiative: access to gene family phylogenies and age distributions
Source: Gigascience. 2020 Feb 11;9(2):giaa004. doi: 10.1093/gigascience/giaa004 (PMC7011446; doi:10.1093/gigascience/giaa004)
Supplement: giaa004_Response_to_Reviewer_Comments_Revision_1 [file giaa004_response_to_reviewer_comments_revision_1.pdf]

Author's Response To Reviewer Comments

Close

Dear Scott ,

Thank you and the reviewer #1 again for all the comments and suggestions on improving our manuscript.

In this revision, we added ORCIDs and RRIDs as you suggested. We added a citation for GigaDB and cited the snapshot of our data in GigaDB. The citation to the newly published 1KP capstone paper is now updated. We also cited the other accompanying GigaScience Data Note in text and mention that the supporting code and sequencing data is described in more detail in the 'Availability of Supporting Data' section.

All changes from the 2nd revision are highlighted in green. Please let me know if you have any questions.

Thanks again,
Li, Zheng

Close
