# Supplementary material for: Inferring putative ancient whole-genome duplications in the 1000 Plants (1KP) initiative: access to gene family phylogenies and age distributions
Source: Gigascience. 2020 Feb 11;9(2):giaa004. doi: 10.1093/gigascience/giaa004 (PMC7011446; doi:10.1093/gigascience/giaa004)
Supplement: giaa004_Reviewer_1_Report_Original_Submission — Yafei Mao -- 8/24/2019 Reviewed [file giaa004_reviewer_1_report_original_submission.pdf]

Reviewer Report

Title: Inferring putative ancient whole genome duplications in the 1000 Plants (1KP) initiative: access to gene family phylogenies and age distributions

Version: Original Submission **Date:** 8/24/2019

Reviewer name: Yafei Mao

Reviewer Comments to Author:

Whole-genome duplications (WGDs) play an important role in the evolution of plants and animals. Investigating the WGDs in plants with 1KP genomic data provides us a big picture of genome evolution in green plants. Although lots of publications have addressed this question, Li's manuscript provides a systematical analysis for the 1KP dataset and then it opens a road for further WGDs' research in the future. Therefore, I recommend this manuscript to publish in the journal.

However, I have a few comments on the analysis and the manuscript's structure.

Major comments:

1. What is the main point in this manuscript? "Building a total evidence approach for WGDs' research" or "Investigating WGDs in green plants with 1KP dataset using a total evidence approach".

The abstract makes me feel that this manuscript's main point on "Investigating WGDs in green plants with 1KP dataset using a total evidence approach", yet the main text makes me feel that the manuscript describes "a total evidence approach" for WGDs' research and using 10KP as a system.

For my understanding, "a total evidence approach" has been applied and described in Jiao's paper and Mao's previous paper (Jiao, Yuannian, et al; Nature; 2011; Mao, Yafei, et al; iScience; 2019). I recommend authors to re-organize the main text to show the point that "Investigating WGDs in green plants with 1KP dataset using a total evidence approach" with INTRODUCTION, METHODS, RESULTS, AND DISCUSSION. This would make this manuscript clear and have much impact on plant WGDs' research in the future.

Plus, a personal taste, I would like to use another word to replace "a total evidence approach", what is the "total evidence"? We might have other methods to infer WGDs and do better in the future. (if authors would like to use "a total evidence approach" in the revised manuscript, I have no problem with it).

2. In abstract, "we release 383,679 nuclear gene family phylogenies and 2,306 gene age distribution (Ks) plots from the 1KP capstone paper." Could you release original data rather plots?

In the method 1.1 "DupPipe analyses of WGDs from transcriptomes of single species", could you release the all orthogroups data which were generated by DupPipe pipeline? As well, in the method 1.3 "MAPS analyses of WGDs from transcriptomes of multiple species", could you release the orthogroups data which were generated by OrthoFinder?

Curious, why do authors use two different methods (DupPipe and OrthoFinder) to generate orthogroups for Ks and MAPS analysis, respectively? (I do not think the methods will have a big impact on the final results, but I'd like to know more about the authors' experimental design.)

3. I am not an expert for statistical tests, I have no more comments on statistical test in this manuscript.

All look good for me by far.
4. In method 1.3, authors used RAxML to reconstruct gene trees, and then I am wondering whether authors used Partitionfinder to select best-fit partitioning schemes as input for RAxML to avoid gene tree bias. If so, authors should mention this in the method section. If not, I recommend the authors to perform this analysis. Alternatively, authors could use IQ-TREE to perform best-fit partitioning schemes finding and tree reconstruction together automatically. My program TREEasy (https://github.com/MaoYafei/TREEasy) might be helpful for a large amount of tree reconstruction with IQ-TREE. However, authors should choose the easier way for themselves to solve this problem, no need to use TREEasy as a must.
5. About Figures, I recommend authors to consider "integrate" Figure 1 and Figure 2 together. If so, it would be more concise for readers. Moreover, If I were authors, I would like to use a table to present Figure 6 (you already have it in your supplementary file).
6. About re-organization of the manuscript as I suggested above, if authors would like to do it, I also suggest that authors could mention WGDs in other clades rather than plants. This might attract more readers who are not studying WGDs in plants.
Minor comments:
1. 1KP_WGD_phylogeny.pdf in (https://bitbucket.org/barkerlab/1kp/src/master/1KP_WGD_phylogeny.pdf) might be truncated. See the top of PDF, some lineages did not show in the pdf file.
2. In "Walk-through examples", like "a median Ks ~0.3". Could you also put the 95% CI there? Also, could you put the line number in the revised manuscript? Sometimes, it is really hard to point out sentences.
3. In "This tendency of our phylogenomic method towards false negatives and "missing" established WGDs is a known issue. There are cases of well-established WGDs going undetected with different phylogenomic analyses, including At-ô�°€[43]". "AT-[]" might have some formatting error here.
4. In "In this project, we used the approach described in Tiley et al. 2018. Previous analyses". Is "in this study" better than "In this project"?
5. Tiley et al. 2018 paper also mentioned that inferred WGDs are more robust when Ks<1 rather than Ks<2. But, I think Ks<2 is acceptable. Maybe it is worth to mention this in the discussion.
6. Using transcriptomes to infer WGD with age distribution sometime is dangerous because transcriptomes assembly mistakes or incomplete transcriptomes assembly would lead to loss of WGD signals. Authors should mention this in the discussion.

Level of Interest

Please indicate how interesting you found the manuscript: Choose an item.

Quality of Written English

Please indicate the quality of language in the manuscript: Choose an item.

Declaration of Competing Interests

Please complete a declaration of competing interests, considering the following questions:

- Have you in the past five years received reimbursements, fees, funding, or salary from an organisation that may in any way gain or lose financially from the publication of this manuscript, either now or in the future?
- Do you hold any stocks or shares in an organisation that may in any way gain or lose financially from the publication of this manuscript, either now or in the future?
- Do you hold or are you currently applying for any patents relating to the content of the manuscript?
- Have you received reimbursements, fees, funding, or salary from an organization that holds or has applied for patents relating to the content of the manuscript?
- Do you have any other financial competing interests?
- Do you have any non-financial competing interests in relation to this paper?

If you can answer no to all of the above, write 'I declare that I have no competing interests' below. If your reply is yes to any, please give details below.

I declare that I have no competing interests

I agree to the open peer review policy of the journal. I understand that my name will be included on my report to the authors and, if the manuscript is accepted for publication, my named report including any attachments I upload will be posted on the website along with the authors' responses. I agree for my report to be made available under an Open Access Creative Commons CC-BY license (http://creativecommons.org/licenses/by/4.0/). I understand that any comments which I do not wish to be included in my named report can be included as confidential comments to the editors, which will not be published.

Choose an item.

To further support our reviewers, we have joined with Publons, where you can gain additional credit to further highlight your hard work (see: https://publons.com/journal/530/gigascience). On publication of this paper, your review will be automatically added to Publons, you can then choose whether or not to claim your Publons credit. I understand this statement.

Yes Choose an item.
