# Supplementary material for: Inferring putative ancient whole-genome duplications in the 1000 Plants (1KP) initiative: access to gene family phylogenies and age distributions
Source: Gigascience. 2020 Feb 11;9(2):giaa004. doi: 10.1093/gigascience/giaa004 (PMC7011446; doi:10.1093/gigascience/giaa004)
Supplement: giaa004_Reviewer_2_Report_Original_Submission — Tao Ma -- 9/4/2019 Reviewed [file giaa004_reviewer_2_report_original_submission.pdf]

Reviewer Report

Title: Inferring putative ancient whole genome duplications in the 1000 Plants (1KP) initiative: access to gene family phylogenies and age distributions

Version: Original Submission **Date:** 9/4/2019

Reviewer name: Tao Ma

Reviewer Comments to Author:

In this manuscript, the authors showed the detailed information about the 1KP data and the pipline they used in the WGD inference. As it is a companion paper, I think it is very necessary and useful to have such a description for the data anslysis.
However, I found that the explanation of the pipline is not very clear for the DupPipe analyses. The authors should have a more detailed description, especially for readers who are not familar with the DupPipe pipeline.
such as:
How they construct gene families using the transcriptomes from only one species?
and How they identify peaks in the distribution of KS values?

Level of Interest

Please indicate how interesting you found the manuscript: Choose an item.

Quality of Written English

Please indicate the quality of language in the manuscript: Choose an item.

Declaration of Competing Interests

Please complete a declaration of competing interests, considering the following questions:

- Have you in the past five years received reimbursements, fees, funding, or salary from an organisation that may in any way gain or lose financially from the publication of this manuscript, either now or in the future?
- Do you hold any stocks or shares in an organisation that may in any way gain or lose financially from the publication of this manuscript, either now or in the future?
- Do you hold or are you currently applying for any patents relating to the content of the manuscript?
- Have you received reimbursements, fees, funding, or salary from an organization that holds or has applied for patents relating to the content of the manuscript?
- Do you have any other financial competing interests?

- Do you have any non-financial competing interests in relation to this paper?

If you can answer no to all of the above, write 'I declare that I have no competing interests' below. If your reply is yes to any, please give details below.

I declare that I have no competing interests

I agree to the open peer review policy of the journal. I understand that my name will be included on my report to the authors and, if the manuscript is accepted for publication, my named report including any attachments I upload will be posted on the website along with the authors' responses. I agree for my report to be made available under an Open Access Creative Commons CC-BY license (http://creativecommons.org/licenses/by/4.0/). I understand that any comments which I do not wish to be included in my named report can be included as confidential comments to the editors, which will not be published.

Choose an item.

To further support our reviewers, we have joined with Publons, where you can gain additional credit to further highlight your hard work (see: https://publons.com/journal/530/gigascience). On publication of this paper, your review will be automatically added to Publons, you can then choose whether or not to claim your Publons credit. I understand this statement.

Yes Choose an item.
